# Overexpression of blueberry *FLOWERING LOCUS T* is associated with changes in the expression of phytohormone-related genes in blueberry plants

**DOI:** 10.1038/hortres.2016.53

**Published:** 2016-10-26

**Authors:** Xuan Gao, Aaron E Walworth, Charity Mackie, Guo-qing Song

**Affiliations:** 1 Department of Horticulture, Plant Biotechnology Resource and Outreach Center, Michigan State University, East Lansing, MI 48824, USA; 2 Key Laboratory for the Conservation and Utilization of Important Biological Resources, College of Life Sciences, Anhui Normal University, Wuhu 241000, China

## Abstract

*Flowering locus T* (*FT*) is a primary integrator in the regulation of plant flowering. Overexpressing a blueberry (*Vaccinium corymbosum* L.) *FT* gene (*VcFT*) (herein *VcFT*-OX) resulted in early flowering and dwarfing in ‘Aurora’ plants (herein ‘VcFT-Aurora’). In this study, we found that *VcFT*-OX reduced shoot regeneration from leaf explants. To investigate the potential roles of the phytohormone pathway genes associated with *VcFT*-OX, differentially expressed (*DE*) genes in leaf tissues of ‘VcFT-Aurora’ plants were annotated and analyzed using non-transgenic ‘Aurora’ plants as a control. Three *DE* floral genes, including the blueberry SUPPRESSOR of Overexpression of constans 1 (*VcSOC1*) (gibberellin related), Abscisic acid responsive elements-binding factor 2 (*VcABF2*) and protein related to ABI3/VP1 (*VcABI3/VP1*) (ethylene-related), are present under both the phytohormone-responsive and the dwarfing-related Gene Ontology terms. The gene networks of the *DE* genes overall showed the molecular basis of the multifunctional aspects of *VcFT* overexpression beyond flowering promotion and suggested that phytohormone changes could be signaling molecules with important roles in the phenotypic changes driven by *VcFT*-OX.

## Introduction

Genetic engineering provides a powerful tool to modify blueberry plants. In our previous studies, we have demonstrated that overexpression of a blueberry (*Vaccinium corymbosum* L.) C-repeat binding factor gene (*VcCBF*) enhances cold tolerance in the southern highbush blueberry cultivar ‘Legacy’; we also identified several functional flowering genes in blueberry, such as *flowering locus T* (*FT*), suppressor of overexpression of constans 1 (*SOC1*), leafy (*LFY*) and apetala1 (*AP1*).^1–3^ Of the flowering pathway genes reported, *FT* is the key integrator of multiple flowering genes that respond to many signals (for example, developmental stage, light, circadian rhythms and temperature);^4^ it promotes plant flowering through the upregulation of its downstream flowering genes (e.g., *SOC1*, *LFY*, and *AP1*). Here, we show that overexpression of a blueberry *FLOWERING LOCUS T* gene (*VcFT*) in the transgenic blueberry cv. Aurora (‘VcFT-Aurora’) was able to drive early and continuous flowering in both *in vitro* shoots and greenhouse-grown 1-year-old plants. In addition, all of the transgenic ‘VcFT-Aurora’ plants displayed dwarf phenotypes.^3^ A similar phenotype has been reported in other *FT*-overexpressing woody plants, such as trifoliate orange,^5^ plum^6^ and hybrid *Eucalyptus* trees.^7^ Early flowering driven by *FT* overexpression is often associated with plant dwarfing.

Florigen was first described in 1937 as a hormone-like molecule that regulates flowering in plants.^8^ The initial florigen hypothesis that ‘flowering would be induced by a specific ratio of known hormones and metabolites’ is not widely accepted, mainly due to the lack of convincing molecular evidence, although phytohormones often have high mobility and stability for long-distance transportation.^8–10^ In 1999, two papers reported the discovery of *FT* in Arabidopsis,^11,12^ which was believed to be Florigen. Functional analyses of *FT* and *FT*-like genes have been reported in numerous studies for 20 plant species of 15 families,^13,14^ including several woody plant species, such as poplar,^15–17^ apple,^18,19^ orange,^5^ grape^20,21^ and blueberry.^3^ The main controversy surrounding the ‘FT-as-florigen’ hypothesis is the low mobility and stability of FT protein for long-distance transport.^22^ Whether the mobile signal of ‘FT-as-florigen’ is not FT protein itself but rather other FT-derivatives with high mobility (for example, phytohormones and low-molecular-weight carbohydrates) remains to be determined.

In general, phytohormones (for example, abscisic acid (ABA), auxin, cytokinin, ethylene, and gibberellins) have important roles in regulating plant development and stature formation. Of these hormones, gibberellic acid (GA) has an important role in regulating plant flowering time and in determining plant stature. Mutations resulting in reduced GA biosynthesis or increased GA degradation often produce dwarf plants with delayed plant flowering.^4,23–26^ Other phytohormone genes (for example, auxin,^27,28^ cytokinin,^10,29,30^ ethylene,^31^ brassinosteroid,^32,33^ jasmonic acid,^34^ nitric oxide,^35^ peptide hormone^36^ and salicylic acid^37,38^) also affect plant flowering and plant size.^9,10^ The mechanisms underlying *FT* overexpression-induced dwarfism are not known.

We developed a blueberry transcriptome reference and identified blueberry flowering pathway genes based on differentially expressed (DE) transcripts in *FT*-overexpressing plants (in comparison with non-transgenic plants).^39^ However, the overall gene networks responding to overexpressing a blueberry (*Vaccinium corymbosum* L.) *FT* gene (*VcFT*) (herein *VcFT*-OX) are not known. The aim of this study was to annotate the blueberry transcriptome reference by using a transcriptome assembly tool called Trinotate (https://trinotate.github.io), to identify the *DE* genes in the phytohormone or dwarfing-related pathways, and to develop the first gene network models in blueberry that show the potential interactions of all DE-expressed genes driven by the *VcFT*-OX. With this research, we hope to reveal all potential roles of phytohormones that underpin the impact of the *VcFT*-OX on plant growth and flowering.

## Materials and methods

### Plant regeneration

A northern highbush blueberry, cv. Aurora, was used. Transgenic ‘Aurora’ plants containing the CaMV 35S-driven *VcFT* were generated in our previous research.^3^ Adventitious shoot regeneration from leaf explants of non-transgenic ‘Aurora’ and one representative transgenic event for both ‘pBISN1-Aurora’ and ‘VcFT-Aurora’ were conducted according to our published protocols.^40,41^ The ‘pBISN1-Aurora’ is a transformation control containing the binary vector pBISN1.^40^ Leaf explants, 10 per petri dish (100×20 mm), were cultured abaxial side up on a 25 ml regeneration medium containing 1.0 mg L^−1^ thidiazuron and 0.5 mg L^−1^ α-naphthaleneacetic acid for 2 weeks in the dark, followed by a 16 h photoperiod of 30 E m^−2^ s^−1^ from cool white fluorescent tubes at 25 °C. Three petri dishes were used as replicates. The number of regenerating explants was recorded after 12 weeks.

For plant phenotyping, 12 plants for non-transgenic events and each of five transgenic events of ‘VcFT-Aurora’ were grown in a secured greenhouse (heated in the winter) under natural light conditions and a regular schedule of irrigation and fertilization using 0.2 g/l fertilizer (Nitrogen:Phosphorus:Potassium=21:7:7).^41^ For full vernalization, 1-year-old plants were grown in the growth chambers at 4 °C with a 12-h photoperiod for 2 months; 2- and 3-year-old plants were exposed to the natural environment in winter in a secured courtyard between our greenhouses. Plant height, flowering time and number of floral buds were recorded.

### RNA preparation and sequencing

Young leaf tissues of 2-year-old ‘Aurora’ and ‘VcFT-Aurora’ plants were collected in June 2014 from the plants that were never exposed to chilling conditions and that showed phenotypic differences in flowering and plant size. Six samples (that is, three ‘Aurora’ plants and three ‘VcFT-Aurora’ plants of one representative transgenic event) were collected, immediately frozen in liquid nitrogen and stored at −80 °C for RNA isolation.

Total RNA was isolated from 0.5 g tissue for each sample using a cetyltrimethylammonium bromide (CTAB) method.^42^ The samples were purified using the RNeasy Mini Kit and On-Column DNase digestion with the RNAse-free DNase Set (Qiagen, Valencia, CA, USA). The integrity of the RNA samples was assessed using the Agilent RNA 6000 Pico Kit (Agilent Technologies, Inc., Santa Clara, CA, USA). All six samples from the three ‘Aurora’ plants and three ‘VcFT-Aurora’ plants of one representative transgenic event had an RNA quality score above 8.0 prior to submission for sequencing and reverse transcription of RNA to complementary DNA (cDNA) for reverse transcription–PCR (RT–PCR). Six cDNA libraries were constructed and two technical replicates for each cDNA library were sequenced in two lanes (100-bp paired end reads) using the Illumina HiSeq2500 platform at the Research Technology Support Facility of Michigan State University (East Lansing, MI, USA).

### *De novo* transcriptome assembly and differential expression analysis

*De novo* transcriptome assembly and differential expression analysis were described in our recent report.^39^ Briefly, RNA sequencing reads of three biological replicates for each of the three non-transgenic ‘Aurora’ plants and three ‘VcFT-Aurora’ plants were analyzed. Two technical replicates were sequenced for each biological replicate. The paired reads, two sets for each biological replicate, were aligned to the transcriptome reference developed for ‘Legacy,’ and the abundance of each read was estimated using the Trinity command ‘align_and_estimate_abundance.pl.’ The Trinity command ‘run_DE_analysis.pl --method edgeR’ was used for differential expression analysis.^43^ The DE genes or transcripts (relative to non-transgenic ‘Aurora’ unless otherwise mentioned) with false discovery rate values below 0.05 were used for further analyses.

### Functional transcriptome annotation and analysis

Annotation of the transcriptome reference and DE transcriptomes (false discovery rate<0.05) of ‘VcFT-Aurora’ was performed using the online Trinotate_v2.0 pipeline (https://trinotate.github.io). All Trinity and Trinotate analyses were performed using the resources at the High Performance Computing Center of Michigan State University. Gene Ontology (GO) slims that contain a subset of GO terms of our annotated transcriptome reference and DE transcriptomes (false discovery rate<0.05) of ‘VcFT-Aurora’ were made by analyzing Top_BLASTP_hits using the Amigo 1.8 tool.

Eleven GO ‘response to 11 phytohormones’ terms and a ‘strigolactone biosynthetic process’ term (GO:0009741) were used to identify genes/transcripts responding to phytohormones from the annotated transcriptome reference (Supplementary Table S1). Additional GO terms were used to identify phytohormone-related DE transcripts in ‘VcFT-Aurora’ (Supplementary Table S1). In addition, the following eight GO terms, derived from previous reports, were used to search for dwarfing-related genes and transcripts: ‘transcription factor activity, sequence-specific DNA binding’ (GO:0003700), ‘multicellular organismal development’ (GO:0007275), ‘response to gibberellin’ (GO:0009739), ‘gibberellic acid mediated signaling pathway’ (GO:0009740), ‘unidimensional cell growth’ (GO:0009826), ‘cell growth’ (GO:0016049), ‘brassinosteroid biosynthetic process’ (GO:0016132) and ‘regulation of timing of transition from vegetative to reproductive phase’ (GO:0048510).^44–51^ Sequence alignment and phylogenetic tree analyses were conducted using CLC Sequence Viewer 7. GO enrichment analysis was conducted from the homepage of the GO Consortium website. Interactive graphs of selected genes were made using BiNGO and DyNet in Cytoscape 3.4.0 (http://www.cytoscape.org).

### Quantitative RT–PCR of DE transcripts

The reliability of DE genes/transcripts identified through RNA sequencing was evaluated through quantitative RT–PCR analysis of 12 selected transcripts (Supplementary Table S2). Reverse transcription of RNA to cDNA was performed using SuperScript II reverse transcriptase (Invitrogen, Carlsbad, CA, USA). The resulting cDNA of 1 μg of RNA was diluted (volume 1:4) in water, and 1 μl/sample (25 ng) was used for each PCR reaction. Three RNA samples from the leaf tissues collected for each of the non-transgenic ‘Aurora’ and transgenic ‘VcFT-Aurora’ lines were used.

The primers were designed using the online tool provided by Integrated DNA Technologies, Inc. (https://www.idtdna.com/Primerquest/Home/Index), where the primers were synthesized (Supplementary Table S2). Quantitative RT–PCR was performed in triplicate on an Agilent Technologies Stratagene Mx3005P (Agilent Technologies) using the SYBR Green system (Life Technologies, Carlsbad, CA, USA). In each 25 μl reaction mixture, 25 ng cDNA, 100 nm primers and 12.5 μl of 2× SYBR Green master mix were included. The reaction conditions for all primer pairs were 95 °C for 10 min, 40 cycles of 30 s at 95 °C, 60 s at 60 °C and 60 s at 72 °C, followed by 1 cycle of 60 s at 95 °C, 30 s at 55 °C and 30 s at 95 °C. The specificity of the application reaction for each primer pair was determined according to the melting curve. Relative expression normalized using the eukaryotic translation initiation factor 3 subunit H was calculated using 2^−ΔΔCt^, where −ΔΔCt=(Ct_GOI_−Ct_nom_)_unknown_−(Ct_GOI_−Ct_nom_)_calibrator_.

## Results

### *VcFT* overexpression reduces plant regeneration frequencies

Transformation with *VcFT* overexpression construct *35S:VcFT* resulted in a lower transformation frequency (2.8% vs 13.3%) compared with that of a GUS reporter construct.^40^ On a regeneration medium without kanamycin selection, all of the leaf explants of non-transgenic ‘Aurora’ and ‘pBISN1-Aurora’ produced multiple shoots (Supplementary Figure S1A); in contrast, only 53.3% (48/90) of the leaf explants of ‘VcFT-Aurora’ had shoot regeneration (Supplementary Figure S1B), suggesting that *VcFT* overexpression in ‘VcFT-Aurora’ has a negative impact on shoot regeneration from the leaf explants.

### Early flowering and dwarfing of *VcFT* overexpressing plants

**Figure 1:**
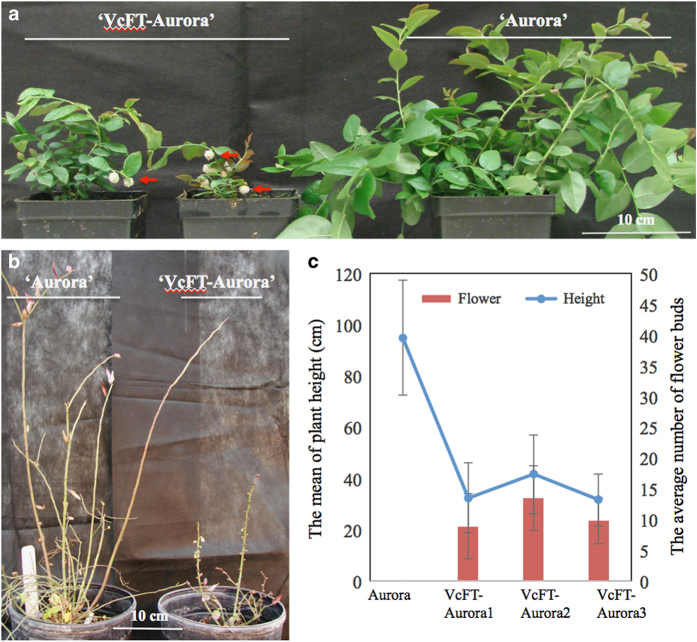
Effect of *VcFT*-OX on plant growth and flowering. (**a**) Flowering of non-vernalized 1-year-old ‘VcFT-Aurora’ (left) alongside ‘Aurora’ plants (right). Arrows show flowers. (**b**) Flowering of fully vernalized 2-year-old ‘Aurora’ (left) alongside ‘VcFT-Aurora’ (right) plants. (**c**) Comparison of plant height (cm) and the number of floral buds in 2-year-old non-transgenic ‘Aurora’ (17 plants) and three transgenic events of ‘VcFT-Aurora’ plants (8–12 plants per event). The plants of the transgenic event VcFT-Aurora1 are the representatives of ‘VcFT-Aurora’.

Similar to plants less than 1-year-old,^3^ 1-year-old ‘VcFT-Aurora’ flowered regardless of vernalization (Figure 1a). The non-transgenic ‘Aurora’ plants did not have floral buds until they were 3 years old (Figure 1b). The vernalized plants flowered with a bloom period of ~1 week, and each bud contained 5–10 flowers, while the unvernalized plants, both non-transgenic and transgenic control ‘pBISNI-Aurora,’ did not flower. In contrast, the plants of all five ‘VcFT-Aurora’ lines showed continuous flowering. In comparison with the vernalized floral buds, unvernalized buds showed a lower percentage (20–50% vs 100%) of flowering buds and a smaller number of flowers (2–3 vs 5–10) in each flowering bud. Under normal growing conditions (that is, full vernalization in winter), the height of 1- to 5-year-old plants of ‘VcFT-Aurora’ was approximately half of that of the non-transgenic ‘Aurora’ (Figures 1a–c). These results indicated that *VcFT* overexpression promotes flowering and reduces plant size, but the need for vernalization is not completely negated.

We also found that ‘VcFT-Aurora’ plants had fewer branches and new shoots than non-transgenic ‘Aurora’ plants. After 4 years of growing without any chilling, neither the ‘VcFT-Aurora’ nor the non-transgenic ‘Aurora’ plants survived. These results indicated that *VcFT*-OX is not sufficient to completely replace the role of vernalization in the normal growth and development of blueberry plants.

### Transcript annotation and GO slims of DE transcripts of the ‘VcFT-Aurora’

Trinotate was used to annotate 3023 DE genes and 4844 DE transcripts identified in ‘VcFT-Aurora,’ and GO terms were assigned to the products of 1991 gene and 4673 transcript contigs. The comparative profiles of the plant GO terms of the DE transcripts of ‘VcFT-Aurora’ revealed a broad impact of *VcFT* overexpression on individual genes and gene networks (Supplementary Figure S2). For example, in the category of biological processes, the top overrepresented GO terms (>30%) were photosynthesis, secondary metabolic processes, post-embryonic development, behavior, anatomical structure and flowering development. At the molecular function level, the top three overrepresented GO terms (>30%) included transcription factor activity and sequence-specific DNA binding, translation regulator activity and RNA binding. The top four overrepresented GO terms (>30%) listed in the category of cellular components were external encapsulating structure, thylakoid, cell wall, and peroxisome. These GO terms show a broad impact of the *VcFT*-OX on plant growth and development (for example, plant size and flowering behaviors).

A total of 267 GO biological process terms (*P*<0.05) were identified from all DE transcripts of the ‘VcFT-Aurora.’ Of these, overrepresented terms included ‘regulation of hormone levels’, ‘developmental process’, ‘reproductive process’, ‘response to stimulus’, ‘signaling’ and ‘nitrogen compound metabolism’. The presence of the GO term ‘regulation of hormone levels’ indicated that phytohormone-related genes are involved in the change in the *VcFT* overexpression plants.

### Pathway genes of the major phytohormones

**Figure 2:**
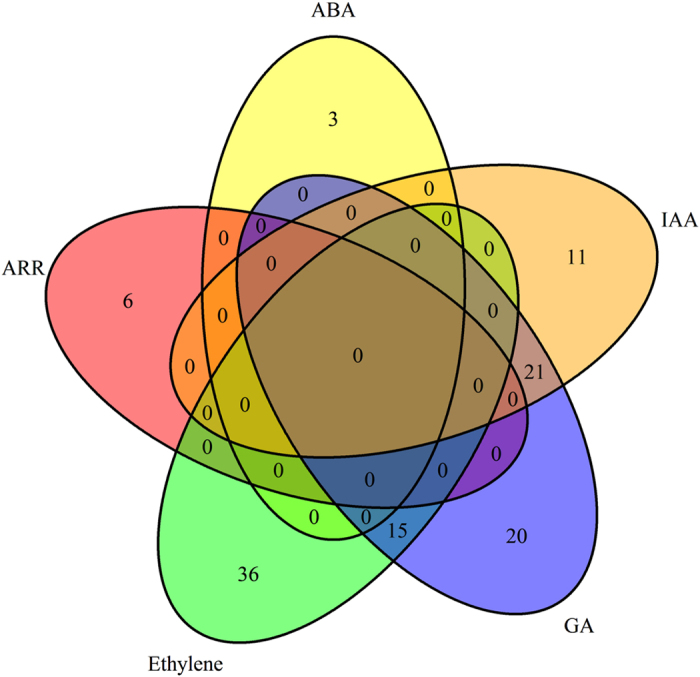
Differentially expressed phytohormone pathway genes in leaf tissue of ‘VcFT-Aurora’ plants.

Of the annotated DE genes, we found 110 pathway genes of five major phytohormones, that is, 3 for ABA, 26 for indole-3-acetic acid (IAA), 6 for cytokinin, 39 for ethylene, and 36 for GA. Of the 36 *DE* genes in the GA pathway, 16 appeared in the IAA pathway and 11 were shared in the ethylene pathway (Figure 2, Table 1). These *DE* genes suggest that the *VcFT*-OX affects a group of phytohormone genes through the regulation of transcript levels.

### Phytohormone-responsive genes/transcripts

**Figure 3:**
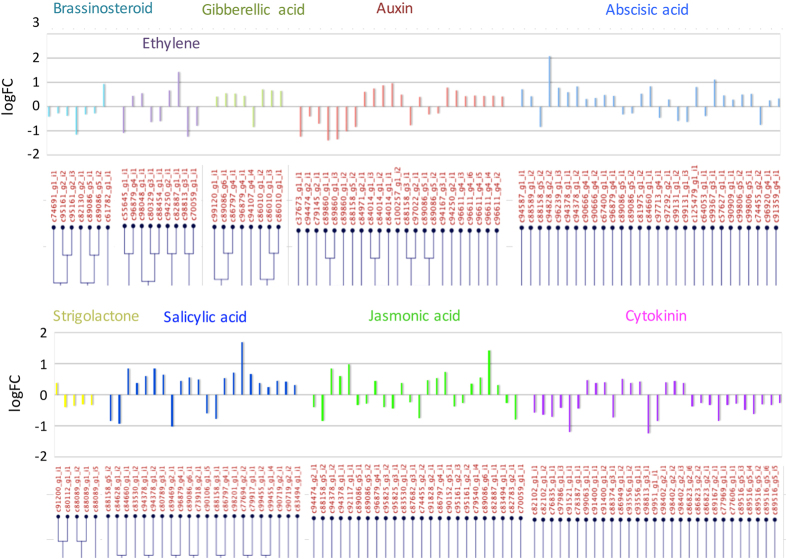
Phytohormone-related differentially expressed transcripts in leaf tissue of ‘VcFT-Aurora’ plants. LogFC: log_2_(fold change)=Log_2_(VcFT-Aurora/Aurora). *X* axis: clusters of transcript identity numbers sorted by genetic distance.

Using the GO terms ‘response to phytohormone-name (that is, abscisic acid, auxin, cytokinin, ethylene, gibberellin, brassinosteroid, jasmonic acid, nitric oxide, peptide hormone and salicylic acid),’ we identified 1571 gene contigs in our blueberry transcriptome reference and 115 genes in the DE transcripts of ‘VcFT-Aurora’ (Figure 3, Table 2, Supplementary Figure S3). Of these 115 DE genes, the genes related to three phytohormones (that is, abscisic acid, gibberellin and salicylic acid) have more upregulated genes than downregulated genes, whereas cytokinin- and brassinosteroid-related genes have more downregulated genes than upregulated genes (Table 2). For example, the GO term ‘response to gibberellin’ was assigned to six *DE* genes, of which five were upregulated and one was downregulated (Figure 3). The highly upregulated DE genes included one ABA-responsive (protein too many mouths), one SA-responsive (wall-associated receptor kinase 2), and one ethylene-responsive (transcription factor *MYB108*). The top two repressed transcripts included one cytokinin-responsive gene (two-component response regulator 1 (*ARR1*)) and one auxin-responsive gene (calcium-binding protein* PBP1*) (Figure 2 and Supplementary Table S2).

To identify all potential phytohormone-related genes beyond those found under the GO terms ‘response to phytohormone-names,’ eight GO terms (Supplementary Table S1) were used to screen the DE transcripts of the ‘VcFT-Aurora.’ We found an additional 31 genes. Of these newly identified transcripts, the top 3 upregulated genes (8.46- to 14.52-fold) are involved in the ABA (1) and ethylene (2) signaling pathways and the top three downregulated genes are related to gibberellin (2) and cytokinin (1) (Supplementary Table S3). In addition, five transcripts of three genes are involved in the strigolactone biosynthetic process (GO:1901601) that functions in plant dwarfing.^52–54^

The phytohormone-related DE transcripts were annotated to 113 known genes (Supplementary Table S3). Of these genes, six flowering genes were identified using the gene/transcript IDs of blueberry flowering genes, including two GA-responsive (*SOC1* and agamous-like 1) genes and one each of the ethylene-responsive (rice early heading date 1 (*OsEhd1*) or *ARR2*), brassinosteroid-responsive (*VcRAV1*), salicylic acid responsive (rice circadian clock associated 1 (*OsCCA1*)), and ABA-activated (*ABF2*) genes. Only the ethylene-responsive (*OsEhd1* or *ARR2*) gene was downregulated, while the others were upregulated. These results reveal the effect of *VcFT* overexpression on the expression of the phytohormone-related genes, which may be potentially responsible for the altered plant growth and development in the ‘VcFT-Aurora’ plants.

### Dwarf-related genes/transcripts

Eight GO terms derived from the dwarf mutants reported in the literature were used to search for dwarf-related genes/transcripts in the DE transcripts of ‘VcFT-Aurora’. A total of 129 DE genes were found (Supplementary Table S4, Supplementary Figure S4). Of these DE genes, we found 12 GA-related genes under GO:0009739 and GO:0009740. Five DE genes were identified under the GO term (GO:0016132) ‘brassinosteroid biosynthetic process’. In addition, *VcFT* was identified under the GO term (GO:0048510) ‘regulation of timing of transition from vegetative to reproductive phase’. Under GO:0003700 ‘transcription factor activity, sequence-specific DNA binding’, we found 146 DE transcripts. The remaining 19 DE transcripts were detected under the GO terms ‘cell growth’, ‘unidimensional cell growth’, and ‘multicellular organismal development’. The importance of GA pathway genes and brassinosteroid pathway genes in dwarf mutants of *Arabidopsis* and rice has been well documented.^44–51,55^

Of the dwarf-related DE transcripts, all 15 *DE* flowering genes, with the exception of *VcFT* (GO:0048510) and *VcLFY* (GO:0007275, multicellular organismal development), were found under the GO term ‘transcription factor activity, sequence-specific DNA binding’. *VcFT*, *VcAP1* and *VcFUL* were the top upregulated genes. *VcAGL19* and *VcOsEhd1* were among the most downregulated genes. *VcSOC1* is GA responsive and upregulated. The involvement of 15 out of 33 DE flowering genes in the DE transcripts related to dwarf plants shows the potential roles of VcFT-OX in affecting both plant flowering and growth.

The majority (166/198) of dwarf-related DE transcript contigs were not among the transcripts of flowering pathway genes. Four highly upregulated transcription factors included OBP3-responsive gene 2 (*ORG2*), ethylene-responsive transcription factor 043 (*ERF043*), dehydration-responsive element-binding protein 3 (*DREB3*), and high mobility group B protein 7 (*HMGB7*). The transcription factor phytoclock 1 (*PCL1*) was the most repressed gene. Of the DREB transcription factors reported, *DREB1E* and *DREB1F* are responsible for *Arabidopsis* mutants deficient in gibberellin biosynthesis.^47^ *ORG2* is annotated as ‘induced by OBF-binding protein 3 (*OBP3*), auxin and salicylic acid, repressed by jasmonic acid, UV light, heat treatments, high iron, low copper and low zinc treatments’.

In general, cell growth contributes to dwarf mutants of *Arabidopsis*.^50,55^ Twelve DE genes were identified under the terms GO:0016049 ‘cell growth’ and GO:0009826 ‘unidimensional cell growth’. Wall-associated receptor kinase 2 (*WAK2*) is the top upregulated gene that may control cell expansion, morphogenesis and development.^56^ The cuticular protein 1 (*CUT1*) is the most downregulated *DE* gene that functions in cuticular wax biosynthesis and pollen fertility.^57^

Of the five GO terms associated with the major dwarf-related DE transcripts in ‘VcFT-Aurora’ (logFC>2^2^ or <2^−2^ fold), the terms ‘response to gibberellin’ and ‘gibberellic acid mediated signaling pathway’ have a direct interaction, and ‘unidimensional cell growth’ interacts with ‘multicellular organismal development’. The term ‘regulation of timing of transition from vegetative to reproductive phase’ does not have any direct interaction with the other two GO term pairs.

### Confirmation of the expression of the selected DE genes

Ten pairs of PCR primers were designed to validate the expression patterns observed in the RNA sequencing for the selected phytohormone- and flowering-related genes (Supplementary Table S2). Our quantitative RT–PCR results confirmed the upregulation (six genes) and downregulation (four genes) of all selected genes (Supplementary Figure S5). In addition to our previous confirmation of the selected DE flowering pathway genes,^39^ our quantitative RT–PCR results gave more evidence to support the reliability of our RNA sequencing data.

### Gene networks of phytohormone-responsive and dwarf-related *DE* genes/transcripts

To visualize the potential interactions, we pooled all phytohormone-related and dwarf-related DE genes/transcripts of ‘VcFT-Aurora’ to identify overrepresented GO terms through BiNGO (*P<*0.05, organism/annotation=*Arabidopsis thaliana*). The gene interactions based on the GOSlim_Plants ontology file displayed 31 overrepresented GO term nodes; for example, under biological process, the terms ‘growth’, ‘flower development’, multicellular organismal development’, ‘carbohydrate metabolic process’, ‘anatomical structure morphogenesis’, ‘response to abiotic stimulus’ and ‘response to endogenous stimulus’ are present (Supplementary Figure S6). This gene network showed the potential biological approaches by which overexpressed *VcFT* may regulate plant growth and development through these phytohormone- and dwarf-related *DE* genes/transcripts of ‘VcFT-Aurora’.

**Figure 4:**
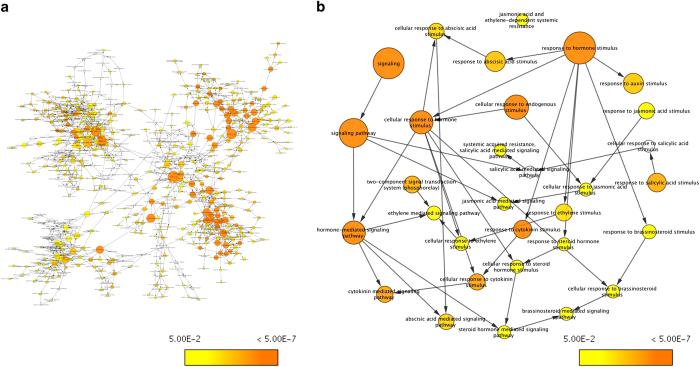
Gene networks of differentially expressed genes (compared with non-transgenic ‘Aurora’) in leaf tissue of transgenic ‘VcFT-Aurora’ plants. (**a**) Gene networks developed using the GO_Biological_Process ontology file in BiNGO for all DE genes. (**b**) Phytohormone-related GO terms in (**a**). Bubble size and color indicate the frequency of the GO term and the *P* value, respectively.

The gene networks of all *DE* genes/transcripts of ‘VcFT-Aurora’ were also developed using both the GOSlim_Plants ontology and biological_process ontology files. In the network based on the GOSlim_Plants ontology, 65 nodes were presented, of which 31 nodes are shared with the network developed using the DE phytohormone- and dwarf-related *DE* genes/transcripts (Supplementary Figure S6B). In the network developed using the biological_process ontology, 599 nodes were presented (Figure 4a), of which 26 phytohormone-related nodes are under the GO term of ‘signaling’ (Figure 4b). More importantly, the presence of phytohormone-related GO terms under the node of ‘signaling pathway’ indicated that phytohormone-signaling is part of the response to *VcFT* overexpression.

## Discussion

Plant flowering in herbaceous plants is generally regulated by the gene networks of *Arabidopsis thaliana* flowering locus C (*FLC*) (vernalization), *CO1* (photoperiod), squamosa promoter-binding-like protein 1 (*SPL1*) (autonomous) and gibberellin 20-oxidase (*GA20ox*) (gibberellin).^4,13,58–67^ *FT* is the main pathway integrator of *FLC* and *CO1*, while *SOC1* is an *FT-*downstream integrator of *GA20ox* and *SPL1*^59,66,68–74^ (<Impact of Wide Hybridization on Highbush blueberry breeding.pdf>). The gibberellin pathways interact with the major floral gene *SOC1* in *A. thaliana*.^75,76^

Over- or ectopic-expression of *FT* and *FT*-like genes can generally cause significant phenotypic changes (for example, shortened plants and precocious and continuous flowering) in plants.^14^ In our recent transcriptome analysis of the *VcFT*-OX transgenic blueberry focusing on flowering pathway genes, 61 transcript contigs of 33 known flowering-related genes showed differential expression.^39^ Of these DE flowering genes, both *VcFT* and *VcSOC1* are major integrators of the flowering pathway. Because *VcSOC1* can interact with GA pathway genes and the ‘VcFT-Aurora’ plants showed retarded growth, we hypothesized that the *VcFT*-OX has a potential impact on retarded plant growth through the expression of either phytohormone genes or dwarf-related genes. Using the annotated RNA sequencing data of our previous samples,^39^ in-depth analysis in this study revealed the overall effect of the *VcFT*-OX on blueberry gene networks (Figure 4).

On the basis of the profiles of *DE* floral genes in ‘VcFT-Aurora’, we propose a *VcFT*-mediated flowering pathway of blueberry, whereby the photoperiod pathway (for example*, VcCOL2* and *VcCOL5*) as well as vernalization and autonomous pathways (for example*, VcFRI, VcMAF2 and VcMAF5*) work through *VcFT* and its downstream integrators (*VcSOC1* and *VcLFY*).^39^ To date, functional *FLC* genes have not been identified in woody plants. In this study, *VcFT*-OX (~2050-fold increase in *VcFT* expression) promoted floral bud formation and flowering, but it did not nullify the need for environmental stimuli for normal flowering; for example, *VcFT* overexpressing ‘VcFT-Aurora’ did not flower normally under no-chilling stress (Figure 1). It appears that there is a *VcFT*-independent pathway in tetraploid blueberry plants through which vernalization modulates plant flowering. More studies are needed to identify those vernalization-responsive genes in blueberry.

**Figure 5:**
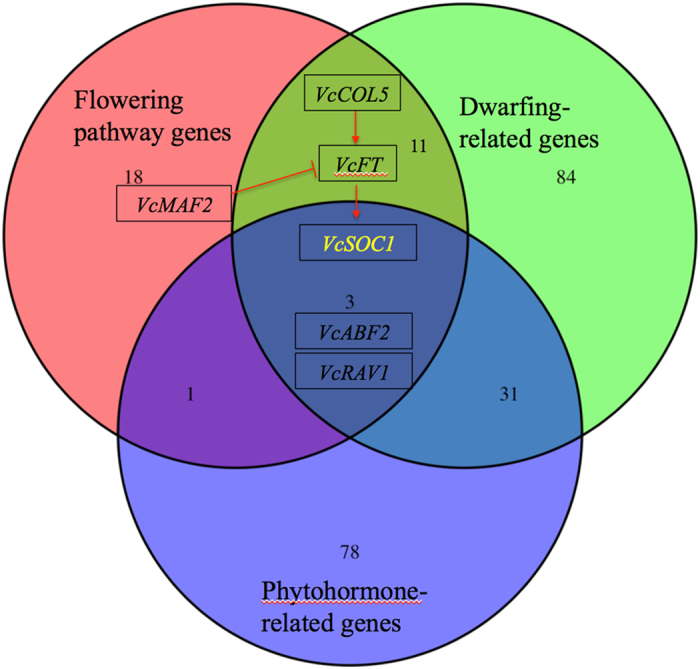
Differentially expressed genes related to plant flowering, phytohormones and plant dwarfing in leaf tissue of ‘VcFT-Aurora’ plants. The background picture shows the phenotypes of a transgenic ‘VcFT-Aurora’ (left) and a non-transgenic ‘Aurora’ plant (right).

The decrease in both the regeneration frequencies of the ‘VcFT-Aurora’ explants and the transformation frequency of the *VcFT* transformation may be due to the altered endogenous phytohormone balance caused by *VcFT* overexpression. Both phytohormone genes and flowering pathway genes can affect plant height and flowering. The retarded growth of the ‘VcFT-Aurora’ plants may also be a by-product of the early transition to a terminating floral meristem due to the flowering promoted by *VcFT*-OX. The reduced vegetative growth in ‘VcFT-Aurora’ may be responsible for the reduced plant size. However, we found interactions of flowering pathway genes with genes in ‘signaling pathway’ (hormone and carbohydrate related), ‘developmental process’ (root and meristem) and ‘regulation of biological process’ (flowering time). In fact, the *VcFT*-OX altered 113 hormone-related genes, of which four are flowering pathway genes (Figure 5). These phytohormone genes appear to have important roles in the simultaneous regulation of plant flowering and plant growth.

Plant size is determined by genetic background and environmental conditions. At the genetic level, several dwarf genes have been reported, including GA pathway genes,^44–46,55^ brassinosteroid pathway genes,^49,51^ transcription factors,^47^ and the F-BOX LEUCINE-TRICH REPEAT PROTEIN (*LRR*) of rice.^50^ The involvement of GA and brassinosteroid pathway genes indicates that both the flowering pathway and phytohormone pathways have impacts on plant size. In this study, the *VcFT*-OX altered 129 dwarf-related genes, of which 14 are flowering pathway genes and 34 are related to phytohormone pathways (Figure 5). Additional studies are needed to determine how these *DE* genes affect plant growth and flowering.

### Conclusion

*VcFT*-OX resulted in differential expression of a total of 110 pathway genes of five major phytohormones, that is, three for ABA, 26 for IAA, six for cytokinin, 39 for ethylene and 36 for GA. Of the 36 *DE* genes in the GA pathway, 16 appear in the IAA pathway and 11 are shared in the ethylene pathway. These *DE* genes in ‘VcFT-Aurora’ plants (versus non-transgenic ‘Aurora’) show the multifunction potential of the overexpressed *VcFT* (Supplementary Figure S2 and Figure 5). For example, as shown in Figure 5, *VcFT* overexpression promoted the expression of *VcSOC1* (GA related), *VcABF2* (ABA related), and *VcRAV1* (ethylene-responsive and brassinosteroid related); these three DE genes are shared in three groups of genes (that is, flowering pathway genes, phytohormone- and dwarf-related genes). The potential interactions of these three groups of genes may be responsible for early flowering and dwarfing in ‘VcFT-Aurora’ plants. The involvement of the pathway genes of five major phytohormones in the ‘VcFT-Aurora’ plants implies that mobile phytohormones may be the signals involved in regulating plant growth and development.

## Figures and Tables

**Table 1:** Summary of phytohormone pathway gene/transcript contigs in blueberry transcriptome reference 'RefTrinity' and differentially expressed (DE) phytohormone pathway gene/transcript contigs in VcFT overexpressing 'VcFT-Aurora

*Phytohormone*	*RefTrinity*		*VcFT overexpression*		*Phytohormone genes of Arabidopsis used for blast*	
	*No. of gene contigs*	*No. of transcript contigs*	*No. of DE gene contigs (upregulated+downregulated)*	*No. of DE transcript contigs (upregulated+downregulated)*	*Number*	*Reference*
ABA	160	329	3 (2+1)	3 (2+1)	14	^77^
Cytokinin^a^	272	6248	6 (5+1)	6 (5+1)	32	^78^
GA	513	3513	36 (26+10)	56 (43+13)	12	^79^
Ethylene	1083	2669	39 (28+11)	51 (36+15)	28	^80^
IAA	398	728	26 (17+9)	33 (22+11)	10	^81^
Sum	2426	13 487	110	149	91	

Abbreviations: ABA, abscisic acid; DE, differentially expressed; GA, gibberellic acid; IAA, indole-3-acetic acid; VcFT, *Vaccinium corymbosum* L. *flowering locus T*.

a Cytokinin was calculated based on the two-component response regulators (*ARRs*).

**Table 2:** Summary of phytohormone-responsive gene/transcript contigs in blueberry transcriptome reference 'RefTrinity' and phytohormone-responsive, differentially expressed (DE) gene/transcript contigs in VcFT overexpressing 'VcFT-Aurora.'

*Phytohormone*	*RefTrinity*		*VcFT overexpression*		*Gene Ontology (GO) term*
	*No. of gene contigs*	*No. of transcript contigs*	*No. of DE gene contigs (upregulated+downregulated)*	*No. of DE transcript contigs (upregulated+downregulated)*	
ABA	460	757	24 (18+6)	29 (21+8)	GO:0009737: response to abscisic acid
Auxins	277	423	14 (7+7)	23 (13+10)	GO:0009733: response to auxin
Cytokinin	215	312	18 (5+13)	29 (9+20)	GO:0009735: response to cytokinin
Ethylene	210	348	9 (4+5)	9 (4+5)	GO:0009723: response to ethylene
Salicylic acid	161	290	19 (14+5)	22 (17+5)	GO:0009751: response to salicylic acid
Jasmonic acid	92	157	20 (9+11)	24 (12+12)	GO:0009753: response to jasmonic acid
Gibberellins	78	156	6 (5+1)	8 (7+1)	GO:0009739: response to gibberellin
Brassinosteroid	71	121	5 (1+4)	7 (1+6)	GO:0009741: response to brassinosteroid
Peptide hormone	4	5	0	0	GO:0043434: response to peptide hormone
Nitric oxide	3	6	0	0	GO:0071731: response to nitric oxide
Polyamines	0	0	0	0	GO:1904583: response to polyamine macromolecule
Strigolactone	0	0	0	0	GO:1902347: response to strigolactone
Sum	1571	2575	115	151	

Abbreviations: ABA, abscisic acid; VcFT, *Vaccinium corymbosum* L. *flowering locus T*.
