# Supplementary material for: Overexpression of blueberry FLOWERING LOCUS T is associated with changes in the expression of phytohormone-related genes in blueberry plants
Source: Hortic Res. 2016 Oct 26;3:16053–. doi: 10.1038/hortres.2016.53 (PMC5080838; doi:10.1038/hortres.2016.53)
Supplement: Supplementary Table S1 [file hortres201653-s1.doc]

| **Table S1** Gene Ontology (GO) terms used to search for genes/transcripts related to phytohormones and dwarf plants | | |
| --- | --- | --- |
| Aspect | ID | Name |
| Process | GO:0009741 | response to brassinosteroid |
| Process | GO:0009723 | response to ethylene |
| Process | GO:0009737 | response to abscisic acid |
| Process | GO:0009733 | response to auxin |
| Process | GO:0009739 | response to gibberellin |
| Process | GO:0009735 | response to cytokinin |
| Process | GO:0071731 | response to nitric oxide |
| Process | GO:0043434 | response to peptide hormone |
| Process | GO:0009751 | response to salicylic acid |
| Process | GO:0009753 | response to jasmonic acid |
| Process | GO:1902347 | response to strigolactone |
|  |  |  |
| Function | GO:0010331 | gibberellin binding |
| Process | GO:0009739 | response to gibberellin |
| Process | GO:0009685 | gibberellin metabolic process |
| Process | GO:0045487 | gibberellin catabolic process |
| Function | GO:0045544 | gibberellin 20-oxidase activity |
| Process | GO:0009686 | gibberellin biosynthetic process |
| Process | GO:0033469 | gibberellin 12 metabolic process |
| Process | GO:0033470 | gibberellin 12 biosynthetic process |
| Function | GO:0047927 | gibberellin-44 dioxygenase activity |
| Process | GO:0010476 | gibberellin mediated signaling pathway |
| Function | GO:0016707 | gibberellin 3-beta-dioxygenase activity |
| Function | GO:0045543 | gibberellin 2-beta-dioxygenase activity |
| Function | GO:0051779 | gibberellin 12-aldehyde oxidase activity |
| Process | GO:0071370 | cellular response to gibberellin stimulus |
| Function | GO:0052634 | C-19 gibberellin 2-beta-dioxygenase activity |
| Function | GO:0052635 | C-20 gibberellin 2-beta-dioxygenase activity |
| Process | GO:0010371 | regulation of gibberellin biosynthetic process |
| Function | GO:0047928 | gibberellin beta-D-glucosyltransferase activity |
| Function | GO:0010341 | gibberellin carboxyl-O-methyltransferase activity |
| Process | GO:0010372 | positive regulation of gibberellin biosynthetic process |
|  |  |  |
| Function | GO:0090411 | brassinosteroid binding |
| Process | GO:0009741 | response to brassinosteroid |
| Process | GO:0010268 | brassinosteroid homeostasis |
| Process | GO:0016131 | brassinosteroid metabolic process |
| Process | GO:0016133 | brassinosteroid catabolic process |
| Process | GO:0016132 | brassinosteroid biosynthetic process |
| Process | GO:0009729 | detection of brassinosteroid stimulus |
| Function | GO:0080118 | brassinosteroid sulfotransferase activity |
| Process | GO:0009742 | brassinosteroid mediated signaling pathway |
| Process | GO:0071367 | cellular response to brassinosteroid stimulus |
| Process | GO:0010422 | regulation of brassinosteroid biosynthetic process |
| Process | GO:1900457 | regulation of brassinosteroid mediated signaling pathway |
| Process | GO:0010423 | negative regulation of brassinosteroid biosynthetic process |
| Process | GO:2000488 | positive regulation of brassinosteroid biosynthetic process |
| Process | GO:1900458 | negative regulation of brassinosteroid mediated signaling pathway |
| Process | GO:1900459 | positive regulation of brassinosteroid mediated signaling pathway |
|  |  |  |
| Process | GO:0010315 | auxin efflux |
| Process | GO:0060919 | auxin influx |
| Function | GO:0010011 | auxin binding |
| Process | GO:0060918 | auxin transport |
| Process | GO:0009733 | response to auxin |
| Process | GO:0010252 | auxin homeostasis |
| Process | GO:0009926 | auxin polar transport |
| Process | GO:0009850 | auxin metabolic process |
| Process | GO:0009852 | auxin catabolic process |
| Function | GO:0038198 | auxin receptor activity |
| Process | GO:0010540 | basipetal auxin transport |
| Process | GO:0010541 | acropetal auxin transport |
| Process | GO:0009851 | auxin biosynthetic process |
| Process | GO:0009721 | detection of auxin stimulus |
| Component | GO:0009921 | auxin efflux carrier complex |
| Process | GO:0080162 | intracellular auxin transport |
| Function | GO:0009672 | auxin:proton symporter activity |
| Process | GO:0009734 | auxin-activated signaling pathway |
| Process | GO:0010249 | auxin conjugate metabolic process |
| Process | GO:0071365 | cellular response to auxin stimulus |
| Process | GO:2000012 | regulation of auxin polar transport |
| Process | GO:0090354 | regulation of auxin metabolic process |
| Process | GO:0010600 | regulation of auxin biosynthetic process |
| Function | GO:0080161 | auxin transmembrane transporter activity |
| Process | GO:0010928 | regulation of auxin mediated signaling pathway |
| Process | GO:0090355 | positive regulation of auxin metabolic process |
| Process | GO:0090356 | negative regulation of auxin metabolic process |
| Function | GO:0010328 | auxin influx transmembrane transporter activity |
| Function | GO:0010329 | auxin efflux transmembrane transporter activity |
| Process | GO:0010601 | positive regulation of auxin biosynthetic process |
| Process | GO:1901703 | protein localization involved in auxin polar transport |
| Process | GO:0010929 | positive regulation of auxin mediated signaling pathway |
| Process | GO:0010930 | negative regulation of auxin mediated signaling pathway |
| Process | GO:0060774 | auxin mediated signaling pathway involved in phyllotactic patterning |
| Process | GO:0090015 | positive regulation of leaflet formation by auxin mediated signaling pathway |
|  |  |  |
| Function | GO:0010331 | gibberellin binding |
| Process | GO:0009739 | response to gibberellin |
| Process | GO:0009685 | gibberellin metabolic process |
| Process | GO:0045487 | gibberellin catabolic process |
| Function | GO:0045544 | gibberellin 20-oxidase activity |
| Process | GO:0009686 | gibberellin biosynthetic process |
| Process | GO:0033469 | gibberellin 12 metabolic process |
| Process | GO:0033470 | gibberellin 12 biosynthetic process |
| Function | GO:0047927 | gibberellin-44 dioxygenase activity |
| Process | GO:0010476 | gibberellin mediated signaling pathway |
| Function | GO:0016707 | gibberellin 3-beta-dioxygenase activity |
| Function | GO:0045543 | gibberellin 2-beta-dioxygenase activity |
| Function | GO:0051779 | gibberellin 12-aldehyde oxidase activity |
| Process | GO:0071370 | cellular response to gibberellin stimulus |
| Function | GO:0052634 | C-19 gibberellin 2-beta-dioxygenase activity |
| Function | GO:0052635 | C-20 gibberellin 2-beta-dioxygenase activity |
| Process | GO:0010371 | regulation of gibberellin biosynthetic process |
| Function | GO:0047928 | gibberellin beta-D-glucosyltransferase activity |
| Function | GO:0010341 | gibberellin carboxyl-O-methyltransferase activity |
| Process | GO:0010372 | positive regulation of gibberellin biosynthetic process |
|  |  |  |
| Function | GO:0090411 | brassinosteroid binding |
| Process | GO:0009741 | response to brassinosteroid |
| Process | GO:0010268 | brassinosteroid homeostasis |
| Process | GO:0016131 | brassinosteroid metabolic process |
| Process | GO:0016133 | brassinosteroid catabolic process |
| Process | GO:0016132 | brassinosteroid biosynthetic process |
| Process | GO:0009729 | detection of brassinosteroid stimulus |
| Function | GO:0080118 | brassinosteroid sulfotransferase activity |
| Process | GO:0009742 | brassinosteroid mediated signaling pathway |
| Process | GO:0071367 | cellular response to brassinosteroid stimulus |
| Process | GO:0010422 | regulation of brassinosteroid biosynthetic process |
| Process | GO:1900457 | regulation of brassinosteroid mediated signaling pathway |
| Process | GO:0010423 | negative regulation of brassinosteroid biosynthetic process |
| Process | GO:2000488 | positive regulation of brassinosteroid biosynthetic process |
| Process | GO:1900458 | negative regulation of brassinosteroid mediated signaling pathway |
| Process | GO:1900459 | positive regulation of brassinosteroid mediated signaling pathway |
|  |  |  |
| Function | GO:0010427 | abscisic acid binding |
| Process | GO:0080168 | abscisic acid transport |
| Process | GO:0009737 | response to abscisic acid |
| Process | GO:1902265 | abscisic acid homeostasis |
| Process | GO:0009687 | abscisic acid metabolic process |
| Process | GO:0046345 | abscisic acid catabolic process |
| Process | GO:0009688 | abscisic acid biosynthetic process |
| Function | GO:0090440 | abscisic acid transporter activity |
| Process | GO:1902266 | cellular abscisic acid homeostasis |
| Process | GO:0009724 | detection of abscisic acid stimulus |
| Function | GO:0010295 | (+)- abscisic acid 8'-hydroxylase activity |
| Function | GO:0010294 | abscisic acid glucosyltransferase activity |
| Process | GO:0071215 | cellular response to abscisic acid stimulus |
| Process | GO:0010115 | regulation of abscisic acid biosynthetic process |
| Function | GO:0051993 | abscisic acid glucose ester beta-glucosidase activity |
| Process | GO:0075343 | modulation by symbiont of abscisic acid levels in host |
| Process | GO:0010116 | positive regulation of abscisic acid biosynthetic process |
| Process | GO:0090359 | negative regulation of abscisic acid biosynthetic process |
| Process | GO:1990218 | positive regulation by symbiont of abscisic acid levels in host |
| Process | GO:1902418 | (+)- abscisic acid D-glucopyranosyl ester transmembrane transport |
| Function | GO:1902417 | (+)- abscisic acid D-glucopyranosyl ester transmembrane transporter activity |
| Process | GO:0009738 | abscisic acid-activated signaling pathway |
| Process | GO:0009787 | regulation of abscisic acid-activated signaling pathway |
| Process | GO:0009788 | negative regulation of abscisic acid-activated signaling pathway |
| Process | GO:0009789 | positive regulation of abscisic acid-activated signaling pathway |
| Process | GO:1901527 | abscisic acid-activated signaling pathway involved in stomatal movement |
|  |  |  |
| Function | GO:0051740 | ethylene binding |
| Process | GO:0009723 | response to ethylene |
| Process | GO:0009692 | ethylene metabolic process |
| Function | GO:0038199 | ethylene receptor activity |
| Process | GO:0042457 | ethylene catabolic process |
| Process | GO:0009693 | ethylene biosynthetic process |
| Process | GO:0009727 | detection of ethylene stimulus |
| Process | GO:0009873 | ethylene-activated signaling pathway |
| Process | GO:0071369 | cellular response to ethylene stimulus |
| Process | GO:0010364 | regulation of ethylene biosynthetic process |
| Function | GO:0038200 | ethylene receptor histidine kinase activity |
| Process | GO:0052021 | modulation by symbiont of ethylene levels in host |
| Process | GO:0010104 | regulation of ethylene-activated signaling pathway |
| Process | GO:0010365 | positive regulation of ethylene biosynthetic process |
| Process | GO:0010366 | negative regulation of ethylene biosynthetic process |
| Process | GO:0075022 | ethylene-mediated activation of appressorium formation |
| Process | GO:0009861 | jasmonic acid and ethylene-dependent systemic resistance |
| Process | GO:1990212 | positive regulation by symbiont of ethylene levels in host |
| Process | GO:0010105 | negative regulation of ethylene-activated signaling pathway |
|  |  |  |
| Biological Process | GO:0009735 | response to cytokinin |
| Process | GO:0009736 | cytokinin-activated signaling pathway |
| Process | GO:0009691 | cytokinin biosynthetic process |
| Process | GO:0009690 | cytokinin metabolic process |
| Process | GO:0009722 | detection of cytokinin stimulus |
| Process | GO:0080037 | negative regulation of cytokinin-activated signaling pathway |
| Process | GO:0071368 | cellular response to cytokinin stimulus |
| Process | GO:0010184 | cytokinin transport |
| Process | GO:0009823 | cytokinin catabolic process |
| Process | GO:0080036 | regulation of cytokinin-activated signaling pathway |
| Function | GO:0044373 | cytokinin binding |
| Function | GO:0009884 | cytokinin receptor activity |
| Function | GO:0001647 | G-protein coupled cytokinin receptor activity |
| Function | GO:0047807 | cytokinin 7-beta-glucosyltransferase activity |
| Function | GO:0080062 | cytokinin 9-beta-glucosyltransferase activity |
| Process | GO:1903856 | regulation of cytokinin dehydrogenase activity |
| Process | GO:1903857 | negative regulation of cytokinin dehydrogenase activity |
| Function | GO:0009885 | transmembrane histidine kinase cytokinin receptor activity |
| Process | GO:1990223 | positive regulation by symbiont of cytokinin levels in host |
| Function | GO:0019139 | cytokinin dehydrogenase activity |
|  |  |  |
| Process | GO:0071731 | response to nitric oxide |
| Process | GO:0071732 | cellular response to nitric oxide |
| Process | GO:0052565 | response to defense-related host nitric oxide production |
| Process | GO:0052569 | obsolete response to defense-related symbiont nitric oxide production |
| Process | GO:0052551 | response to defense-related nitric oxide production by other organism involved in symbiotic interaction |
| Process | GO:0052060 | evasion or tolerance by symbiont of host-produced nitric oxide |
| Process | GO:0052375 | obsolete evasion or tolerance by organism of symbiont-produced nitric oxide |
| Process | GO:0052376 | evasion or tolerance by organism of nitric oxide produced by other organism involved in symbiotic interaction |
| Process | GO:0051409 | response to nitrosative stress |
| Process | GO:0071500 | cellular response to nitrosative stress |
| Process | GO:1990442 | intrinsic apoptotic signaling pathway in response to nitrosative stress |
| Process | GO:0061403 | positive regulation of transcription from RNA polymerase II promoter in response to nitrosative stress |
| Process | GO:0002537 | nitric oxide production involved in inflammatory response |
| Process | GO:0052063 | induction by symbiont of defense-related host nitric oxide production |
| Process | GO:0052163 | modulation by symbiont of defense-related host nitric oxide production |
| Process | GO:0052347 | positive regulation by symbiont of defense-related host nitric oxide production |
| Process | GO:0052395 | obsolete induction by organism of defense-related symbiont nitric oxide production |
| Process | GO:0052457 | obsolete modulation by organism of defense-related symbiont nitric oxide production |
| Process | GO:0052346 | obsolete positive regulation by organism of defense-related symbiont nitric oxide production |
| Process | GO:0052263 | induction by organism of defense-related nitric oxide production in other organism involved in symbiotic interaction |
|  |  |  |
| Process | GO:1904583 | response to polyamine macromolecule |
| Process | GO:1904584 | cellular response to polyamine macromolecule |
|  |  |  |
| Function | GO:1901149 | salicylic acid binding |
| Process | GO:0009751 | response to salicylic acid |
| Process | GO:0009696 | salicylic acid metabolic process |
| Process | GO:0046244 | salicylic acid catabolic process |
| Process | GO:0009697 | salicylic acid biosynthetic process |
| Process | GO:0009752 | detection of salicylic acid stimulus |
| Process | GO:0009863 | salicylic acid mediated signaling pathway |
| Process | GO:0071446 | cellular response to salicylic acid stimulus |
| Process | GO:0010337 | regulation of salicylic acid metabolic process |
| Process | GO:0080142 | regulation of salicylic acid biosynthetic process |
| Process | GO:0052023 | modulation by symbiont of salicylic acid levels in host |
| Process | GO:2000031 | regulation of salicylic acid mediated signaling pathway |
| Function | GO:0052639 | salicylic acid glucosyltransferase (ester-forming) activity |
| Function | GO:0052640 | salicylic acid glucosyltransferase (glucoside-forming) activity |
| Process | GO:0080151 | positive regulation of salicylic acid mediated signaling pathway |
| Process | GO:1990213 | negative regulation by symbiont of salicylic acid levels in host |
| Process | GO:0052468 | obsolete modulation by organism of salicylic acid levels in symbiont |
| Process | GO:0009862 | systemic acquired resistance, salicylic acid mediated signaling pathway |
| Process | GO:0010679 | cinnamic acid biosynthetic process involved in salicylic acid metabolism |
| Process | GO:0052469 | modulation by organism of salicylic acid levels in other organism involved in symbiotic interaction |
|  |  |  |
| Process | GO:0009867 | jasmonic acid mediated signaling pathway |
| Process | GO:0009753 | response to jasmonic acid |
