# Supplementary material for: Overexpression of blueberry FLOWERING LOCUS T is associated with changes in the expression of phytohormone-related genes in blueberry plants
Source: Hortic Res. 2016 Oct 26;3:16053–. doi: 10.1038/hortres.2016.53 (PMC5080838; doi:10.1038/hortres.2016.53)
Supplement: Supplementary Table S2 [file hortres201653-s2.doc]

**Table S2** Primers used for RT-PCR. FDR (false discovery rate) = 0.05. LogFC: log_2_(fold change) =Log_2_(VcFT-Aurora/Aurora).

| Primer name | Primer (5' to 3' end) | Gene ID | Contig | LogFC | FDR |
| --- | --- | --- | --- | --- | --- |
| GA_F | ATG CTC TCT TGT TGC TTA TTC C | GA | c89469_g2_i1 | -2.063 | 0.000 |
| GA_R | CTT GTT GGG TCT TCC AGT TAT T | GA | c89469_g2_i1 | -2.063 | 0.000 |
| PBP1_F | TCG TGT TCG AGG ACT TCT TT | PBP1 | c89860_g1_i1 | -2.033 | 0.054 |
| ERF43_F | GCT ACT ACT GCA ACA CTT CAT C | ERF43 | c91057_g4_i1 | 3.614 | 0.000 |
| ERF43_R | TTC CTT GGC TCC CTG ATT T | ERF43 | c91057_g4_i1 | 3.614 | 0.000 |
| KAO2_F | CCA ACA TGA TGA GGT TAG AGA A | KAO2 | C92839_g2_i5 | 4.941 | 0.000 |
| KAO2_R | GGA GAC TGA GTG AGG TAG AA | KAO2 | C92839_g2_i5 | 4.941 | 0.000 |
| PBP1_R | CAA CTG ATT CAA CTC GCC ATC | PBP1 | c89860_g1_i1 | -2.033 | 0.054 |
| EFE_F | GCC TCC ATT CCT TCT AAC TAC | EFE | c93875_g1_i4 | 1.175 | 0.000 |
| EFE_R | CTT CCA ATC CTA AGC CCT TC | EFE | c93875_g1_i4 | 1.175 | 0.000 |
| SEPALLATA 1_F | CGG TTG GAG AAG ATG ATG AGA G | Developmental protein SEPALLATA 1 | c88116_g6_i1 | 2.360 | 1 |
| SEPALLATA 1_F | AGA GTG GAG TTG AAG AGG ATA GA | Developmental protein SEPALLATA 1 | c88116_g6_i1 | 2.360 | 1 |
| COL 5_F | ACG CTA TCC GAT CCA GAA TTG | Zinc finger protein CONSTANS-LIKE 5 | c85121_g2_i1 | -1.371 | 0.254 |
| COL5_R | CGA TGC TCC GGA GAT GAA AT | Zinc finger protein CONSTANS-LIKE 5 | c85121_g2_i1 | -1.371 | 0.254 |
| SOC1_F | TTC CCT TCT CTC TCT CTC TCT C | MADS-box protein SOC1 | c86010_g1_i2 | 1.430 | 0.000 |
| SOC1_R | CAC TCT CCT CCT CCT CCT ATA A | MADS-box protein SOC1 | c86010_g1_i2 | 1.430 | 0.000 |
| ARR2_F | CAG CCT TAT CAA TCC CGA GTT | Two-component resonse regulator ARR2 | c98813_g3_i1 | -2.483 | 0.004 |
| ARR2_R | ATG ATC CAT CCA CAG CAA AGA | Two-component response regulator ARR2 | c98813_g3_i1 | -2.483 | 0.004 |
| ABA hydroxylase 4_F | AGA CAA GAC AGA TGC CGA TTA C | Abscisic acid 8’-hydroxylase 4 | c83950_g1_i2 | 1.908 | 0.000 |
| ABA hydroxylase 4_R | GTC TAC TGC CTC TCT GAA TGT G | Abscisic acid 8’-hydroxylase 4 | c83950_g1_i2 | 1.908 | 0.000 |
| EIF_F | CAG AAG AGA AGG GCT GAG AAC | Eukaryotic translation initiation factor 3 subunit H | c82677_g1_i1 | 0 | 1 |
| EIF_R | GGT TCA GGG ATC GGC TTA AA | Eukaryotic translation initiation factor 3 subunit H | c82677_g1_i1 | 0 | 1 |
