# Supplementary material for: Overexpression of blueberry FLOWERING LOCUS T is associated with changes in the expression of phytohormone-related genes in blueberry plants
Source: Hortic Res. 2016 Oct 26;3:16053–. doi: 10.1038/hortres.2016.53 (PMC5080838; doi:10.1038/hortres.2016.53)
Supplement: Supplementary Table S3 [file hortres201653-s3.doc]

| **Table S3** DE phytohormone-related genes (compared to non-transgenic 'Aurora') in leaf tissues of 'VcFT-Aurora'. LogFC: log_2_(fold change) =Log_2_(VcFT-Aurora/Aurora) | | | | | | | |
| --- | --- | --- | --- | --- | --- | --- | --- |
| #gene_id | transcript_id | sprot_Top_BLASTP_hit | Phytohormone | logFC | logCPM | PValue | FDR |
| c99096_g4 | c99096_g4_i1 | AB2C_ARATH | (+)- abscisic acid D-glucopyranosyl ester transmembrane transport | 1.037 | 1.439 | 0.001 | 0.038 |
| c78662_g1 | c78662_g1_i1 | . | abscisic acid binding | -2.419 | 2.888 | 0.000 | 0.000 |
| c81454_g2 | c81454_g2_i3 | GCR2_ARATH | abscisic acid binding | -1.015 | 1.112 | 0.001 | 0.026 |
| c83567_g1 | c83567_g1_i1 | PYL9_ARATH | abscisic acid binding | -0.733 | 3.293 | 0.000 | 0.023 |
| c80869_g3 | c80869_g3_i1 | ABAH4_ARATH | abscisic acid catabolic process | -2.928 | -1.374 | 0.000 | 0.004 |
| c80869_g3 | c80869_g3_i2 | ABAH3_ORYSJ | abscisic acid catabolic process | -2.101 | -0.561 | 0.001 | 0.028 |
| c80869_g3 | c80869_g3_i6 | ABAH4_ARATH | abscisic acid catabolic process | -1.537 | -0.163 | 0.001 | 0.031 |
| c83950_g1 | c83950_g1_i1 | ABAH4_ARATH | abscisic acid catabolic process | -1.557 | 3.018 | 0.000 | 0.001 |
| c83950_g1 | c83950_g1_i2 | ABAH4_ARATH | abscisic acid catabolic process | -1.908 | 2.801 | 0.000 | 0.000 |
| c99120_g1 | c99120_g1_i1 | XERIC_ARATH | abscisic acid metabolic process | -0.835 | 5.384 | 0.000 | 0.000 |
| c84537_g1 | c84537_g1_i1 | CDPK3_ARATH | abscisic acid-activated signaling pathway | -0.488 | 6.009 | 0.000 | 0.023 |
| c85724_g1 | c85724_g1_i1 | AI5L5_ARATH | abscisic acid-activated signaling pathway | -0.698 | 4.849 | 0.000 | 0.003 |
| c97085_g4 | c97085_g4_i1 | P2C07_ARATH | abscisic acid-activated signaling pathway | -0.635 | 4.999 | 0.000 | 0.000 |
| c97085_g4 | c97085_g4_i2 | P2C07_ARATH | abscisic acid-activated signaling pathway | -0.803 | 5.025 | 0.000 | 0.000 |
| c97085_g4 | c97085_g4_i4 | P2C07_ARATH | abscisic acid-activated signaling pathway | -0.771 | 4.563 | 0.000 | 0.000 |
| c97085_g4 | c97085_g4_i5 | P2C07_ARATH | abscisic acid-activated signaling pathway | -0.667 | 3.592 | 0.000 | 0.007 |
| c79724_g2 | c79724_g2_i1 | YUC6_ARATH | auxin biosynthetic process | -0.923 | 2.482 | 0.000 | 0.007 |
| c82097_g1 | c82097_g1_i1 | YUC2_ARATH | auxin biosynthetic process | -0.800 | 2.239 | 0.001 | 0.043 |
| c89516_g5 | c89516_g5_i2 | TRPA2_ARATH | auxin biosynthetic process | 0.632 | 4.736 | 0.000 | 0.000 |
| c89516_g5 | c89516_g5_i3 | TRPA2_ARATH | auxin biosynthetic process | 0.971 | 4.430 | 0.000 | 0.000 |
| c89516_g5 | c89516_g5_i4 | TRPA2_ARATH | auxin biosynthetic process | 1.231 | 3.145 | 0.000 | 0.000 |
| c89516_g5 | c89516_g5_i5 | TRPA2_ARATH | auxin biosynthetic process | 0.519 | 4.559 | 0.000 | 0.013 |
| c89516_g5 | c89516_g5_i6 | TRPA2_ARATH | auxin biosynthetic process | 0.660 | 4.594 | 0.000 | 0.000 |
| c89516_g5 | c89516_g5_i7 | TRPA1_ARATH | auxin biosynthetic process | 1.064 | 4.853 | 0.000 | 0.000 |
| c92695_g1 | c92695_g1_i1 | WAT1_ARATH | auxin biosynthetic process | -0.853 | 7.215 | 0.000 | 0.008 |
| c87750_g5 | c87750_g5_i1 | RHM1_ARATH | auxin efflux | -0.939 | 1.828 | 0.000 | 0.009 |
| c57449_g1 | c57449_g1_i1 | PIN8_ARATH | auxin homeostasis | 1.190 | 1.736 | 0.000 | 0.002 |
| c90563_g2 | c90563_g2_i1 | AUX1_ARATH | auxin polar transport | 1.173 | 2.662 | 0.000 | 0.000 |
| c90563_g3 | c90563_g3_i1 | LAX2_MEDTR | auxin polar transport | 0.743 | 3.445 | 0.000 | 0.016 |
| c95840_g3 | c95840_g3_i1 | PIN3_ARATH | auxin polar transport | 0.506 | 7.334 | 0.000 | 0.001 |
| c95840_g3 | c95840_g3_i2 | PIN3_ARATH | auxin polar transport | 0.523 | 7.367 | 0.000 | 0.002 |
| c44634_g1 | c44634_g1_i1 | LAX4_MEDTR | auxin-activated signaling pathway | 1.825 | -0.215 | 0.000 | 0.007 |
| c63793_g1 | c63793_g1_i1 | AX15A_SOYBN | auxin-activated signaling pathway | 0.836 | 4.953 | 0.000 | 0.000 |
| c75341_g1 | c75341_g1_i2 | LAX4_MEDTR | auxin-activated signaling pathway | 1.360 | 2.043 | 0.000 | 0.000 |
| c80149_g1 | c80149_g1_i1 | . | auxin-activated signaling pathway | -2.609 | -0.281 | 0.000 | 0.000 |
| c81845_g1 | c81845_g1_i1 | AXX15_SOYBN | auxin-activated signaling pathway | 0.906 | 4.033 | 0.000 | 0.000 |
| c81911_g1 | c81911_g1_i1 | GSTF_HYOMU | auxin-activated signaling pathway | 0.722 | 6.593 | 0.000 | 0.000 |
| c83311_g3 | c83311_g3_i1 | IAA30_ORYSJ | auxin-activated signaling pathway | -0.612 | 3.176 | 0.000 | 0.013 |
| c87099_g1 | c87099_g1_i1 | PID2_ORYSJ | auxin-activated signaling pathway | -1.017 | 3.512 | 0.000 | 0.000 |
| c87099_g2 | c87099_g2_i1 | PID2_ARATH | auxin-activated signaling pathway | -1.218 | 3.744 | 0.000 | 0.000 |
| c89177_g2 | c89177_g2_i1 | 12KD_FRAAN | auxin-activated signaling pathway | -1.130 | 6.178 | 0.000 | 0.000 |
| c89177_g2 | c89177_g2_i2 | 12KD_FRAAN | auxin-activated signaling pathway | -1.012 | 5.761 | 0.000 | 0.000 |
| c96198_g2 | c96198_g2_i1 | . | auxin-activated signaling pathway | 1.279 | 1.880 | 0.000 | 0.000 |
| c74422_g1 | c74422_g1_i1 | . | basipetal auxin transport | -1.121 | 4.001 | 0.000 | 0.000 |
| c86600_g2 | c86600_g2_i1 | ZIFL1_ARATH | basipetal auxin transport | -0.566 | 4.295 | 0.001 | 0.031 |
| c92831_g1 | c92831_g1_i1 | SPHK1_ARATH | cellular response to abscisic acid stimulus | 0.621 | 5.062 | 0.000 | 0.000 |
| c94978_g2 | c94978_g2_i1 | PUB9_ARATH | cellular response to abscisic acid stimulus | -1.130 | 5.020 | 0.000 | 0.000 |
| c74280_g1 | c74280_g1_i1 | LOG7_ARATH | cytokinin biosynthetic process | 1.919 | 2.295 | 0.000 | 0.000 |
| c74280_g1 | c74280_g1_i2 | LOG3_ARATH | cytokinin biosynthetic process | 1.288 | 2.615 | 0.000 | 0.000 |
| c80278_g2 | c80278_g2_i1 | LOG1_ARATH | cytokinin biosynthetic process | 1.541 | 1.686 | 0.000 | 0.000 |
| c80278_g2 | c80278_g2_i2 | LOG3_ARATH | cytokinin biosynthetic process | 1.207 | 2.200 | 0.000 | 0.000 |
| c80278_g2 | c80278_g2_i3 | LOG1_ARATH | cytokinin biosynthetic process | 1.844 | 1.483 | 0.000 | 0.000 |
| c55095_g1 | c55095_g1_i1 | ZFP6_ARATH | cytokinin-activated signaling pathway | -1.566 | 0.166 | 0.001 | 0.028 |
| c79824_g2 | c79824_g2_i1 | AHP1_ARATH | cytokinin-activated signaling pathway | -0.592 | 3.778 | 0.000 | 0.009 |
| c82793_g2 | c82793_g2_i1 | AHP3_ARATH | cytokinin-activated signaling pathway | 0.972 | 3.132 | 0.000 | 0.000 |
| c82793_g2 | c82793_g2_i2 | AHP1_ARATH | cytokinin-activated signaling pathway | 0.785 | 3.881 | 0.000 | 0.000 |
| c82793_g2 | c82793_g2_i4 | . | cytokinin-activated signaling pathway | 0.946 | 3.271 | 0.000 | 0.000 |
| c82793_g2 | c82793_g2_i5 | . | cytokinin-activated signaling pathway | 0.784 | 1.869 | 0.001 | 0.048 |
| c82793_g2 | c82793_g2_i6 | . | cytokinin-activated signaling pathway | 0.996 | 1.296 | 0.000 | 0.017 |
| c86949_g1 | c86949_g1_i1 | ARR8_ARATH | cytokinin-activated signaling pathway | -1.078 | 1.452 | 0.000 | 0.018 |
| c61782_g1 | c61782_g1_i1 | RAV1_ARATH | ethylene-activated signaling pathway | -1.872 | 0.764 | 0.000 | 0.018 |
| c74782_g1 | c74782_g1_i1 | WIN1_ARATH | ethylene-activated signaling pathway | -1.180 | 2.472 | 0.000 | 0.000 |
| c82783_g2 | c82783_g2_i1 | CUL1_ARATH | ethylene-activated signaling pathway | 0.538 | 3.607 | 0.001 | 0.030 |
| c82913_g1 | c82913_g1_i1 | RA212_ARATH | ethylene-activated signaling pathway | -0.652 | 7.717 | 0.000 | 0.001 |
| c85019_g1 | c85019_g1_i1 | ERF17_ARATH | ethylene-activated signaling pathway | 1.580 | 0.824 | 0.000 | 0.007 |
| c86546_g2 | c86546_g2_i1 | ERF25_ARATH | ethylene-activated signaling pathway | 1.838 | 1.367 | 0.000 | 0.000 |
| c90612_g1 | c90612_g1_i1 | ERF61_ARATH | ethylene-activated signaling pathway | -1.025 | 3.943 | 0.000 | 0.000 |
| c90612_g1 | c90612_g1_i2 | ERF61_ARATH | ethylene-activated signaling pathway | -0.986 | 3.833 | 0.000 | 0.000 |
| c91032_g1 | c91032_g1_i1 | EF118_ARATH | ethylene-activated signaling pathway | -0.670 | 6.364 | 0.000 | 0.010 |
| c91032_g1 | c91032_g1_i2 | EF118_ARATH | ethylene-activated signaling pathway | -0.697 | 6.031 | 0.000 | 0.001 |
| c91057_g4 | c91057_g4_i1 | ERF43_ARATH | ethylene-activated signaling pathway | -3.614 | 1.071 | 0.000 | 0.000 |
| c91057_g4 | c91057_g4_i2 | ERF43_ARATH | ethylene-activated signaling pathway | -2.838 | -0.725 | 0.000 | 0.001 |
| c91057_g4 | c91057_g4_i4 | ERF43_ARATH | ethylene-activated signaling pathway | -3.079 | 0.439 | 0.000 | 0.000 |
| c94474_g2 | c94474_g2_i1 | CUL1_ARATH | ethylene-activated signaling pathway | 0.814 | 2.478 | 0.000 | 0.023 |
| c97220_g2 | c97220_g2_i2 | MOCOS_SOLLC | ethylene-activated signaling pathway | -0.716 | 2.349 | 0.001 | 0.044 |
| c55095_g1 | c55095_g1_i1 | ZFP6_ARATH | gibberellic acid mediated signaling pathway | -1.566 | 0.166 | 0.001 | 0.028 |
| c76098_g1 | c76098_g1_i1 | . | gibberellic acid mediated signaling pathway | 0.520 | 5.145 | 0.000 | 0.018 |
| c83856_g1 | c83856_g1_i2 | GID1B_ARATH | gibberellic acid mediated signaling pathway | -0.863 | 3.078 | 0.000 | 0.002 |
| c89469_g2 | c89469_g2_i1 | . | gibberellic acid mediated signaling pathway | 2.063 | 1.340 | 0.000 | 0.000 |
| c92766_g2 | c92766_g2_i1 | GAT21_ARATH | gibberellic acid mediated signaling pathway | 0.537 | 3.840 | 0.000 | 0.018 |
| c92984_g1 | c92984_g1_i1 | KO1_ARATH | gibberellic acid mediated signaling pathway | -1.302 | 2.772 | 0.000 | 0.003 |
| c92984_g1 | c92984_g1_i2 | KO1_ARATH | gibberellic acid mediated signaling pathway | -1.281 | 1.954 | 0.001 | 0.049 |
| c92984_g1 | c92984_g1_i3 | KO1_ARATH | gibberellic acid mediated signaling pathway | -1.422 | 1.397 | 0.000 | 0.001 |
| c70648_g1 | c70648_g1_i1 | G3OX_PEA | gibberellin biosynthetic process | -1.371 | 0.353 | 0.001 | 0.026 |
| c85645_g3 | c85645_g3_i2 | G2OX1_PEA | gibberellin biosynthetic process | -1.322 | 1.568 | 0.000 | 0.001 |
| c85645_g3 | c85645_g3_i3 | G2OX1_PEA | gibberellin biosynthetic process | -1.959 | 0.157 | 0.000 | 0.000 |
| c92984_g1 | c92984_g1_i1 | KO1_ARATH | gibberellin biosynthetic process | -1.302 | 2.772 | 0.000 | 0.003 |
| c92984_g1 | c92984_g1_i2 | KO1_ARATH | gibberellin biosynthetic process | -1.281 | 1.954 | 0.001 | 0.049 |
| c96791_g4 | c96791_g4_i1 | KSA_PEA | gibberellin biosynthetic process | 2.450 | 1.593 | 0.000 | 0.000 |
| c95825_g3 | c95825_g3_i1 | COI1_ARATH | jasmonic acid and ethylene-dependent systemic resistance | 0.887 | 3.335 | 0.000 | 0.000 |
| c82783_g2 | c82783_g2_i1 | CUL1_ARATH | jasmonic acid mediated signaling pathway | 0.538 | 3.607 | 0.001 | 0.030 |
| c82783_g2 | c82783_g2_i1 | CUL1_ARATH | jasmonic acid mediated signaling pathway | 0.538 | 3.607 | 0.001 | 0.030 |
| c94474_g2 | c94474_g2_i1 | CUL1_ARATH | jasmonic acid mediated signaling pathway | 0.814 | 2.478 | 0.000 | 0.023 |
| c94474_g2 | c94474_g2_i1 | CUL1_ARATH | jasmonic acid mediated signaling pathway | 0.814 | 2.478 | 0.000 | 0.023 |
| c95161_g2 | c95161_g2_i2 | C90B1_ARATH | jasmonic acid mediated signaling pathway | 0.553 | 4.085 | 0.001 | 0.045 |
| c95161_g2 | c95161_g2_i3 | C90B1_ARATH | jasmonic acid mediated signaling pathway | 0.762 | 3.548 | 0.000 | 0.012 |
| c95825_g3 | c95825_g3_i1 | COI1_ARATH | jasmonic acid mediated signaling pathway | 0.887 | 3.335 | 0.000 | 0.000 |
| c95825_g3 | c95825_g3_i2 | COI1_ARATH | jasmonic acid mediated signaling pathway | 0.797 | 3.167 | 0.000 | 0.003 |
| c96611_g4 | c96611_g4_i2 | TPL_ARATH | jasmonic acid mediated signaling pathway | -0.841 | 5.344 | 0.000 | 0.000 |
| c96611_g4 | c96611_g4_i3 | TPL_ARATH | jasmonic acid mediated signaling pathway | -0.868 | 5.388 | 0.000 | 0.000 |
| c96611_g4 | c96611_g4_i4 | TPL_ARATH | jasmonic acid mediated signaling pathway | -0.906 | 5.434 | 0.000 | 0.000 |
| c96611_g4 | c96611_g4_i5 | TPL_ARATH | jasmonic acid mediated signaling pathway | -0.884 | 5.477 | 0.000 | 0.000 |
| c96611_g4 | c96611_g4_i6 | TPL_ARATH | jasmonic acid mediated signaling pathway | -0.921 | 5.373 | 0.000 | 0.000 |
| c97220_g2 | c97220_g2_i2 | MOCOS_SOLLC | jasmonic acid mediated signaling pathway | -0.716 | 2.349 | 0.001 | 0.044 |
| c95953_g1 | c95953_g1_i1 | P2C03_ARATH | negative regulation of abscisic acid-activated signaling pathway | -3.863 | 1.404 | 0.000 | 0.000 |
| c96116_g3 | c96116_g3_i1 | P2C03_ARATH | negative regulation of abscisic acid-activated signaling pathway | -1.127 | 5.575 | 0.000 | 0.000 |
| c91546_g2 | c91546_g2_i1 | PLDD1_ARATH | positive regulation of abscisic acid-activated signaling pathway | -1.520 | 1.125 | 0.000 | 0.001 |
| c91546_g2 | c91546_g2_i2 | PLDD1_ARATH | positive regulation of abscisic acid-activated signaling pathway | -1.200 | 3.565 | 0.000 | 0.000 |
| c79875_g1 | c79875_g1_i1 | FBX6_ARATH | regulation of auxin mediated signaling pathway | 1.217 | 1.080 | 0.000 | 0.006 |
| c96959_g1 | c96959_g1_i1 | ICR1_ARATH | regulation of auxin polar transport | -1.482 | 0.689 | 0.000 | 0.002 |
| c96959_g1 | c96959_g1_i2 | ICR1_ARATH | regulation of auxin polar transport | -0.754 | 2.651 | 0.001 | 0.035 |
| c96959_g1 | c96959_g1_i3 | ICR1_ARATH | regulation of auxin polar transport | -0.851 | 4.461 | 0.000 | 0.003 |
| 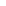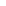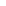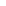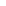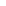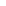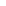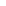   | c125479_g1 | | --- | | c125479_g1_i1 | IP5P3_ARATH | response to abscisic acid | -1.625 | 0.950 | 0.001 | 0.032 |
| c57627_g1 | c57627_g1_i1 | COR47_ARATH | response to abscisic acid | -0.932 | 5.451 | 0.000 | 0.000 |
| c64053_g1 | c64053_g1_i1 | ALFC2_ARATH | response to abscisic acid | 0.784 | 4.410 | 0.000 | 0.000 |
| c67400_g1 | c67400_g1_i1 | . | response to abscisic acid | -0.969 | 6.197 | 0.000 | 0.000 |
| c74455_g2 | c74455_g2_i2 | . | response to abscisic acid | 1.513 | -0.461 | 0.001 | 0.043 |
| c81269_g1 | c81269_g1_i1 | CML24_ARATH | response to abscisic acid | 1.509 | 4.178 | 0.000 | 0.004 |
| c81975_g1 | c81975_g1_i2 | LTI65_ARATH | response to abscisic acid | -1.072 | 0.964 | 0.001 | 0.033 |
| c84660_g1 | c84660_g1_i1 | MYB3_ARATH | response to abscisic acid | -1.681 | 1.967 | 0.000 | 0.008 |
| c88158_g5 | c88158_g5_i2 | . | response to abscisic acid | 1.680 | 2.671 | 0.000 | 0.000 |
| c89086_g5 | c89086_g5_i2 | OAT_ORYSJ | response to abscisic acid | 0.560 | 4.276 | 0.000 | 0.006 |
| c90666_g4 | c90666_g4_i1 | HA22D_ARATH | response to abscisic acid | -0.636 | 4.837 | 0.000 | 0.002 |
| c90909_g1 | c90909_g1_i1 | KEG_ARATH | response to abscisic acid | -0.577 | 4.491 | 0.000 | 0.002 |
| c94378_g1 | c94378_g1_i1 | PTR3_ARATH | response to abscisic acid | -1.195 | 1.912 | 0.000 | 0.000 |
| c94828_g2 | c94828_g2_i2 | TMM_ARATH | response to abscisic acid | -4.171 | -1.615 | 0.000 | 0.001 |
| c96239_g1 | c96239_g1_i3 | FBW2_ARATH | response to abscisic acid | -1.562 | 0.644 | 0.000 | 0.003 |
| c96920_g4 | c96920_g4_i1 | RSH2C_ARATH | response to abscisic acid | -0.517 | 4.981 | 0.001 | 0.036 |
| c97292_g2 | c97292_g2_i1 | PARP1_ARATH | response to abscisic acid | -0.595 | 4.684 | 0.000 | 0.009 |
| c97713_g4 | c97713_g4_i1 | . | response to abscisic acid | 0.927 | 4.207 | 0.000 | 0.000 |
| c99131_g1 | c99131_g1_i2 | PP370_ARATH | response to abscisic acid | 1.188 | 1.291 | 0.000 | 0.003 |
| c99131_g1 | c99131_g1_i3 | PP370_ARATH | response to abscisic acid | 1.267 | 1.514 | 0.000 | 0.000 |
| c99367_g3 | c99367_g3_i1 | RFS5_ARATH | response to abscisic acid | -2.234 | -0.283 | 0.000 | 0.001 |
| c99806_g5 | c99806_g5_i1 | AB11G_ARATH | response to abscisic acid | -1.059 | 4.981 | 0.000 | 0.000 |
| c99806_g5 | c99806_g5_i2 | AB11G_ARATH | response to abscisic acid | -1.004 | 4.514 | 0.000 | 0.001 |
| c100257_g1 | c100257_g1_i2 | SNC1_ARATH | response to auxin | -0.997 | 4.995 | 0.000 | 0.000 |
| c37673_g1 | c37673_g1_i1 | AB1B_ARATH | response to auxin | 2.481 | -1.217 | 0.001 | 0.039 |
| c79145_g2 | c79145_g2_i1 | PBP1_ARATH | response to auxin | 1.411 | 2.679 | 0.000 | 0.000 |
| c84014_g1 | c84014_g1_i1 | IAA14_ARATH | response to auxin | -1.931 | 1.556 | 0.000 | 0.000 |
| c84014_g1 | c84014_g1_i2 | IAA14_ARATH | response to auxin | -1.763 | 1.328 | 0.000 | 0.000 |
| c84014_g1 | c84014_g1_i3 | IAA14_ARATH | response to auxin | -1.503 | 1.355 | 0.000 | 0.004 |
| c84971_g2 | c84971_g2_i1 | IAA14_ARATH | response to auxin | -1.228 | 4.121 | 0.000 | 0.000 |
| c88158_g3 | c88158_g3_i1 | BT1_ARATH | response to auxin | 1.545 | 5.700 | 0.000 | 0.000 |
| c89860_g1 | c89860_g1_i1 | PBP1_ARATH | response to auxin | 2.033 | -1.030 | 0.001 | 0.054 |
| c89860_g1 | c89860_g1_i2 | . | response to auxin | 2.703 | -0.059 | 0.000 | 0.000 |
| c89860_g1 | c89860_g1_i3 | PBP1_ARATH | response to auxin | 2.792 | 0.181 | 0.000 | 0.000 |
| c94167_g3 | c94167_g3_i1 | ARFE_ARATH | response to auxin | -1.578 | 4.776 | 0.000 | 0.000 |
| c96611_g4 | c96611_g4_i2 | TPL_ARATH | response to auxin | -0.841 | 5.344 | 0.000 | 0.000 |
| c96611_g4 | c96611_g4_i3 | TPL_ARATH | response to auxin | -0.868 | 5.388 | 0.000 | 0.000 |
| c96611_g4 | c96611_g4_i4 | TPL_ARATH | response to auxin | -0.906 | 5.434 | 0.000 | 0.000 |
| c96611_g4 | c96611_g4_i5 | TPL_ARATH | response to auxin | -0.884 | 5.477 | 0.000 | 0.000 |
| c96611_g4 | c96611_g4_i6 | TPL_ARATH | response to auxin | -0.921 | 5.373 | 0.000 | 0.000 |
| |  |  |  |  | | --- | --- | --- | --- | |  | 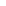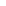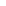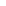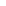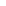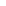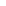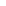 |  | 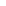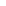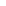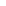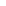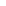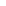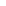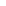 |  | c97022_g2 | | --- | | c97022_g2_i1 | F3PH_ARATH | response to auxin | -0.805 | 3.714 | 0.000 | 0.015 |
| c61782_g1 | c61782_g1_i1 | RAV1_ARATH | response to brassinosteroid | -1.872 | 0.764 | 0.000 | 0.018 |
| c74691_g1 | c74691_g1_i1 | IBH1_ORYSJ | response to brassinosteroid | 0.830 | 3.735 | 0.000 | 0.000 |
| c82130_g2 | c82130_g2_i1 | PME41_ARATH | response to brassinosteroid | 2.309 | 0.411 | 0.000 | 0.000 |
| c89086_g5 | c89086_g5_i1 | OAT_ORYSJ | response to brassinosteroid | 0.648 | 3.930 | 0.000 | 0.003 |
| c89086_g5 | c89086_g5_i2 | OAT_ORYSJ | response to brassinosteroid | 0.560 | 4.276 | 0.000 | 0.006 |
| c95161_g2 | c95161_g2_i2 | C90B1_ARATH | response to brassinosteroid | 0.553 | 4.085 | 0.001 | 0.045 |
| c95161_g2 | c95161_g2_i3 | C90B1_ARATH | response to brassinosteroid | 0.762 | 3.548 | 0.000 | 0.012 |
| c76835_g1 | c76835_g1_i1 | CP41A_ARATH | response to cytokinin | 1.440 | 0.413 | 0.000 | 0.008 |
| c77606_g1 | c77606_g1_i1 | RK5_ARATH | response to cytokinin | 0.570 | 7.989 | 0.000 | 0.000 |
| c77969_g1 | c77969_g1_i1 | CB4B_ARATH | response to cytokinin | 0.646 | 10.738 | 0.000 | 0.015 |
| c78387_g1 | c78387_g1_i1 | RK15_ARATH | response to cytokinin | 0.899 | 8.888 | 0.000 | 0.000 |
| c82102_g1 | c82102_g1_i1 | CB4B_ARATH | response to cytokinin | 1.144 | 3.075 | 0.000 | 0.000 |
| c82102_g1 | c82102_g1_i2 | CB4B_ARATH | response to cytokinin | 1.285 | 2.905 | 0.000 | 0.000 |
| c83885_g1 | c83885_g1_i1 | TL16_ARATH | response to cytokinin | 0.461 | 6.724 | 0.001 | 0.027 |
| c86823_g2 | c86823_g2_i1 | LPA3_ARATH | response to cytokinin | 0.647 | 3.321 | 0.000 | 0.019 |
| c86823_g2 | c86823_g2_i6 | LPA3_ARATH | response to cytokinin | 0.757 | 2.466 | 0.000 | 0.019 |
| c88374_g3 | c88374_g3_i1 | . | response to cytokinin | 1.456 | 0.223 | 0.000 | 0.011 |
| c89167_g2 | c89167_g2_i1 | ACR11_ARATH | response to cytokinin | 1.667 | -0.121 | 0.001 | 0.042 |
| c91400_g1 | c91400_g1_i1 | ENO1_ARATH | response to cytokinin | -0.769 | 5.446 | 0.001 | 0.026 |
| c91400_g1 | c91400_g1_i2 | ENO1_ARATH | response to cytokinin | -0.810 | 5.394 | 0.000 | 0.015 |
| c91521_g1 | c91521_g1_i1 | APA1_ARATH | response to cytokinin | 2.387 | -0.988 | 0.000 | 0.006 |
| c93556_g1 | c93556_g1_i1 | ARR11_ARATH | response to cytokinin | -0.866 | 3.672 | 0.000 | 0.001 |
| c97986_g1 | c97986_g1_i1 | NDUAC_ARATH | response to cytokinin | 1.293 | 5.938 | 0.000 | 0.000 |
| c97986_g1 | c97986_g1_i3 | NDUAC_ARATH | response to cytokinin | 0.849 | 4.009 | 0.000 | 0.000 |
| c98402_g2 | c98402_g2_i1 | 4CLLA_ARATH | response to cytokinin | -0.800 | 6.417 | 0.000 | 0.001 |
| c98402_g2 | c98402_g2_i2 | 4CLLA_ARATH | response to cytokinin | -0.889 | 6.255 | 0.000 | 0.000 |
| c98402_g2 | c98402_g2_i3 | 4CLLA_ARATH | response to cytokinin | -0.764 | 6.737 | 0.000 | 0.000 |
| c99063_g1 | c99063_g1_i1 | ACC1_ARATH | response to cytokinin | -0.955 | 8.759 | 0.000 | 0.004 |
| c9951_g1 | c9951_g1_i1 | RK15_ARATH | response to cytokinin | 1.703 | 0.547 | 0.000 | 0.000 |
| c55645_g1 | c55645_g1_i1 | HMA7_ARATH | response to ethylene | 2.175 | -1.168 | 0.001 | 0.032 |
| c70059_g1 | c70059_g1_i1 | ACBP4_ARATH | response to ethylene | 1.587 | 0.094 | 0.000 | 0.008 |
| c80329_g3 | c80329_g3_i1 | RMA1_CAPAN | response to ethylene | 1.277 | 3.037 | 0.000 | 0.000 |
| c82887_g1 | c82887_g1_i1 | MY108_ARATH | response to ethylene | -2.864 | -1.421 | 0.001 | 0.042 |
| c83494_g1 | c83494_g1_i1 | MYB44_ARATH | response to ethylene | -0.636 | 2.800 | 0.001 | 0.041 |
| c88454_g1 | c88454_g1_i1 | RMA1_CAPAN | response to ethylene | 1.207 | 3.578 | 0.000 | 0.000 |
| c94250_g2 | c94250_g2_i1 | MYB12_ARATH | response to ethylene | -1.341 | 3.002 | 0.000 | 0.000 |
| c96879_g4 | c96879_g4_i1 | RVE6_ARATH | response to ethylene | -0.889 | 4.895 | 0.000 | 0.000 |
| c98048_g1 | c98048_g1_i1 | TAR2_ARATH | response to ethylene | -1.110 | 2.377 | 0.000 | 0.000 |
| c98813_g3 | c98813_g3_i1 | ARR2_ARATH | response to ethylene | 2.483 | -0.907 | 0.000 | 0.004 |
| c83856_g1 | c83856_g1_i2 | GID1B_ARATH | response to gibberellin | -0.863 | 3.078 | 0.000 | 0.002 |
| c86010_g1 | c86010_g1_i1 | SOC1_ARATH | response to gibberellin | -1.297 | 4.487 | 0.000 | 0.000 |
| c86010_g1 | c86010_g1_i2 | SOC1_ARATH | response to gibberellin | -1.429 | 4.165 | 0.000 | 0.000 |
| c86010_g1 | c86010_g1_i3 | SOC1_ARATH | response to gibberellin | -1.340 | 4.510 | 0.000 | 0.000 |
| c86797_g4 | c86797_g4_i1 | . | response to gibberellin | -1.082 | 1.789 | 0.000 | 0.024 |
| 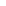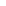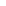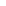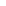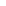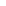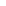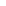   | c89086_g6 | | --- | | c89086_g6_i1 | MYB28_ARATH | response to gibberellin | -1.107 | 5.416 | 0.000 | 0.000 |
| c70059_g1 | c70059_g1_i1 | ACBP4_ARATH | response to jasmonic acid | 1.587 | 0.094 | 0.000 | 0.008 |
| c74455_g2 | c74455_g2_i2 | . | response to jasmonic acid | 1.513 | -0.461 | 0.001 | 0.043 |
| c79540_g1 | c79540_g1_i4 | MY113_ARATH | response to jasmonic acid | -0.697 | 2.855 | 0.001 | 0.030 |
| c82887_g1 | c82887_g1_i1 | MY108_ARATH | response to jasmonic acid | -2.864 | -1.421 | 0.001 | 0.042 |
| c83494_g1 | c83494_g1_i1 | MYB44_ARATH | response to jasmonic acid | -0.636 | 2.800 | 0.001 | 0.041 |
| c83530_g1 | c83530_g1_i2 | WRK70_ARATH | response to jasmonic acid | -0.752 | 3.232 | 0.000 | 0.012 |
| c86797_g4 | c86797_g4_i1 | . | response to jasmonic acid | -1.082 | 1.789 | 0.000 | 0.024 |
| c87682_g3 | c87682_g3_i1 | GMPP1_ARATH | response to jasmonic acid | 0.477 | 6.089 | 0.000 | 0.004 |
| c88158_g5 | c88158_g5_i2 | . | response to jasmonic acid | 1.680 | 2.671 | 0.000 | 0.000 |
| c89086_g5 | c89086_g5_i1 | OAT_ORYSJ | response to jasmonic acid | 0.648 | 3.930 | 0.000 | 0.003 |
| c89086_g5 | c89086_g5_i2 | OAT_ORYSJ | response to jasmonic acid | 0.560 | 4.276 | 0.000 | 0.006 |
| c89086_g6 | c89086_g6_i1 | MYB28_ARATH | response to jasmonic acid | -1.107 | 5.416 | 0.000 | 0.000 |
| c90152_g5 | c90152_g5_i1 | SOT15_ARATH | response to jasmonic acid | -1.454 | 1.259 | 0.000 | 0.000 |
| c91828_g2 | c91828_g2_i1 | ILL6_ARATH | response to jasmonic acid | -0.940 | 3.347 | 0.001 | 0.036 |
| c92117_g1 | c92117_g1_i1 | PTR27_ARATH | response to jasmonic acid | -1.937 | 0.872 | 0.000 | 0.000 |
| c94378_g1 | c94378_g1_i1 | PTR3_ARATH | response to jasmonic acid | -1.195 | 1.912 | 0.000 | 0.000 |
| c94378_g1 | c94378_g1_i2 | PTR3_ARATH | response to jasmonic acid | -1.671 | 1.055 | 0.000 | 0.000 |
| c95161_g2 | c95161_g2_i2 | C90B1_ARATH | response to jasmonic acid | 0.553 | 4.085 | 0.001 | 0.045 |
| c95161_g2 | c95161_g2_i3 | C90B1_ARATH | response to jasmonic acid | 0.762 | 3.548 | 0.000 | 0.012 |
| c95825_g3 | c95825_g3_i1 | COI1_ARATH | response to jasmonic acid | 0.887 | 3.335 | 0.000 | 0.000 |
| c95825_g3 | c95825_g3_i2 | COI1_ARATH | response to jasmonic acid | 0.797 | 3.167 | 0.000 | 0.003 |
| c96879_g4 | c96879_g4_i1 | RVE6_ARATH | response to jasmonic acid | -0.889 | 4.895 | 0.000 | 0.000 |
| c73918_g2 | c73918_g2_i1 | MYB46_ARATH | response to salicylic acid | -0.996 | 1.045 | 0.001 | 0.029 |
| c77694_g2 | c77694_g2_i2 | WAK2_ARATH | response to salicylic acid | -3.393 | -1.479 | 0.000 | 0.006 |
| c79917_g1 | c79917_g1_i1 | RDR1_ARATH | response to salicylic acid | -1.315 | 2.637 | 0.000 | 0.000 |
| c80789_g3 | c80789_g3_i1 | RDR1_ARATH | response to salicylic acid | -1.292 | 2.250 | 0.000 | 0.000 |
| c83494_g1 | c83494_g1_i1 | MYB44_ARATH | response to salicylic acid | -0.636 | 2.800 | 0.001 | 0.041 |
| c83530_g1 | c83530_g1_i2 | WRK70_ARATH | response to salicylic acid | -0.752 | 3.232 | 0.000 | 0.012 |
| c84628_g1 | c84628_g1_i2 | GSTU7_ARATH | response to salicylic acid | 1.863 | 1.779 | 0.000 | 0.000 |
| c84660_g1 | c84660_g1_i1 | MYB3_ARATH | response to salicylic acid | -1.681 | 1.967 | 0.000 | 0.008 |
| c86797_g4 | c86797_g4_i1 | . | response to salicylic acid | -1.082 | 1.789 | 0.000 | 0.024 |
| c86797_g4 | c86797_g4_i1 | . | response to salicylic acid | -1.082 | 1.789 | 0.000 | 0.024 |
| c88158_g3 | c88158_g3_i1 | BT1_ARATH | response to salicylic acid | 1.545 | 5.700 | 0.000 | 0.000 |
| c88158_g5 | c88158_g5_i2 | . | response to salicylic acid | 1.680 | 2.671 | 0.000 | 0.000 |
| c89086_g6 | c89086_g6_i1 | MYB28_ARATH | response to salicylic acid | -1.107 | 5.416 | 0.000 | 0.000 |
| c89086_g6 | c89086_g6_i1 | MYB28_ARATH | response to salicylic acid | -1.107 | 5.416 | 0.000 | 0.000 |
| c89469_g2 | c89469_g2_i1 | . | response to salicylic acid | 2.063 | 1.340 | 0.000 | 0.000 |
| c90106_g1 | c90106_g1_i5 | WAK2_ARATH | response to salicylic acid | 1.206 | 2.343 | 0.000 | 0.000 |
| c90719_g2 | c90719_g2_i1 | RDR1_ARATH | response to salicylic acid | -0.911 | 2.560 | 0.000 | 0.003 |
| c90719_g2 | c90719_g2_i2 | RDR1_ARATH | response to salicylic acid | -0.831 | 1.939 | 0.001 | 0.034 |
| c94378_g1 | c94378_g1_i1 | PTR3_ARATH | response to salicylic acid | -1.195 | 1.912 | 0.000 | 0.000 |
| c94378_g1 | c94378_g1_i2 | PTR3_ARATH | response to salicylic acid | -1.671 | 1.055 | 0.000 | 0.000 |
| c96879_g4 | c96879_g4_i1 | RVE6_ARATH | response to salicylic acid | -0.889 | 4.895 | 0.000 | 0.000 |
| c98201_g1 | c98201_g1_i1 | LRK41_ARATH | response to salicylic acid | -1.413 | 6.662 | 0.000 | 0.000 |
| c99455_g1 | c99455_g1_i2 | RDR1_ARATH | response to salicylic acid | -0.733 | 3.869 | 0.000 | 0.002 |
| c99455_g1 | c99455_g1_i4 | RDR1_ARATH | response to salicylic acid | -0.504 | 5.257 | 0.001 | 0.029 |
| c80112_g1 | c80112_g1_i1 | DAD2_PETHY | strigolactone biosynthetic process | 0.822 | 5.642 | 0.000 | 0.002 |
| c88089_g1 | c88089_g1_i1 | D27_ORYSJ | strigolactone biosynthetic process | 0.622 | 5.295 | 0.001 | 0.033 |
| c88089_g1 | c88089_g1_i2 | D27_ORYSJ | strigolactone biosynthetic process | 0.731 | 5.269 | 0.000 | 0.003 |
| c88089_g1 | c88089_g1_i5 | D27_ORYSJ | strigolactone biosynthetic process | 0.672 | 4.909 | 0.000 | 0.019 |
| c91200_g1 | c91200_g1_i1 | D14_ORYSJ | strigolactone biosynthetic process | -0.769 | 6.995 | 0.000 | 0.005 |
