# Supplementary material for: Overexpression of blueberry FLOWERING LOCUS T is associated with changes in the expression of phytohormone-related genes in blueberry plants
Source: Hortic Res. 2016 Oct 26;3:16053–. doi: 10.1038/hortres.2016.53 (PMC5080838; doi:10.1038/hortres.2016.53)
Supplement: Supplementary Table 4 [file hortres201653-s4.doc]

| 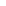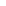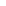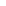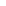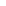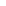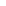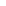**Table 4** Defferentially expressed dwarf-related genes in 'VcFT-Aurora' (vs. nontransgenic 'Aurora'). FDR (false discovery rate) = 0.05. LogFC: log_2_(fold change) =Log_2_(VcFT-Aurora/Aurora). *Transcript has more than one annotation | | | | | | | | |
| --- | --- | --- | --- | --- | --- | --- | --- | --- |
| Gene_id | Transcript_id | logFC | logCPM: log2(counts per million) | PValue | FDR | Sprot_Top_BLASTP_hit | Gene Ontology term | Flowering gene |
| c89084_g2 | c89084_g2_i1 | -3.552 | -2.270 | 3.05E-20 | 6.90E-17 | PCLL_ARATH | GO:0003700:transcription factor activity, sequence-specific DNA binding |  |
| c51453_g1 | c51453_g1_i1 | -3.526 | 2.075 | 0.000640085 | 0.030731511 | . | GO:0009826:unidimensional cell growth |  |
| c89084_g2 | c89084_g2_i3 | -3.186 | -2.152 | 2.17E-17 | 3.54E-14 | APL_ARATH | GO:0003700:transcription factor activity, sequence-specific DNA binding |  |
| c98813_g3 | c98813_g3_i1 | -2.483 | 0.907 | 4.97E-05 | 0.004251957 | ARR2_ARATH | GO:0003700:transcription factor activity, sequence-specific DNA binding | OsEhd1 |
| c96791_g4 | c96791_g4_i1 | -2.45 | -1.593 | 4.94E-12 | 3.30E-09 | KSA_PEA | GO:0009739:response to gibberellin |  |
| c90432_g6 | c90432_g6_i1 | -2.428 | -0.279 | 9.17E-09 | 2.93E-06 | BZP43_ARATH | GO:0003700:transcription factor activity, sequence-specific DNA binding |  |
| c89469_g2 | c89469_g2_i1 | -2.063 | -1.340 | 2.59E-08 | 7.49E-06 | . | GO:0009740:gibberellic acid mediated signaling pathway |  |
| c86546_g2 | c86546_g2_i1 | -1.838 | -1.367 | 1.43E-10 | 7.30E-08 | ERF25_ARATH | GO:0003700:transcription factor activity, sequence-specific DNA binding |  |
| c94107_g4 | c94107_g4_i4 | -1.695 | -1.171 | 1.57E-06 | 0.00025228 | SOC1_ARATH | GO:0003700:transcription factor activity, sequence-specific DNA binding | AGL19 |
| c97106_g3 | *c97106_g3_i3 | -1.69 | 0.589 | 0.000949271 | 0.041127828 | HFA7B_ARATH | GO:0003700:transcription factor activity, sequence-specific DNA binding |  |
| c97106_g3 | *c97106_g3_i3 | -1.69 | 0.589 | 0.000949271 | 0.041127828 | HFA6B_ARATH | GO:0003700:transcription factor activity, sequence-specific DNA binding |  |
| c12861_g1 | c12861_g1_i1 | -1.594 | -1.898 | 1.57E-07 | 3.57E-05 | HD1_ORYSJ | GO:0003700:transcription factor activity, sequence-specific DNA binding |  |
| c85019_g1 | c85019_g1_i1 | -1.58 | -0.824 | 8.95E-05 | 0.006730663 | ERF17_ARATH | GO:0003700:transcription factor activity, sequence-specific DNA binding |  |
| c92265_g1 | c92265_g1_i2 | -1.457 | -0.984 | 4.40E-06 | 0.000596341 | ZFP4_ARATH | GO:0003700:transcription factor activity, sequence-specific DNA binding |  |
| c53141_g1 | c53141_g1_i1 | -1.419 | -0.774 | 3.47E-05 | 0.003183551 | COL3_ARATH | GO:0003700:transcription factor activity, sequence-specific DNA binding |  |
| c74760_g1 | c74760_g1_i1 | -1.329 | -1.962 | 0.000122495 | 0.00857351 | . | GO:0007275:multicellular organismal development |  |
| c92265_g1 | c92265_g1_i1 | -1.243 | -2.949 | 3.36E-11 | 1.94E-08 | ZFP4_ARATH | GO:0003700:transcription factor activity, sequence-specific DNA binding |  |
| c92182_g2 | c92182_g2_i2 | -1.23 | -2.331 | 7.87E-08 | 1.99E-05 | TCP19_ARATH | GO:0003700:transcription factor activity, sequence-specific DNA binding | CHE |
| c90106_g1 | c90106_g1_i5 | -1.206 | -2.343 | 1.37E-07 | 3.18E-05 | WAK2_ARATH | GO:0009826:unidimensional cell growth |  |
| c81577_g5 | c81577_g5_i1 | -1.164 | -0.816 | 0.000813735 | 0.036682641 | GAT28_ARATH | GO:0003700:transcription factor activity, sequence-specific DNA binding |  |
| c66990_g1 | c66990_g1_i1 | -1.16 | -0.729 | 0.000783814 | 0.035620468 | NAC86_ARATH | GO:0003700:transcription factor activity, sequence-specific DNA binding |  |
| c92265_g1 | c92265_g1_i3 | -1.157 | -3.174 | 1.90E-10 | 9.34E-08 | ZFP4_ARATH | GO:0003700:transcription factor activity, sequence-specific DNA binding |  |
| c85121_g1 | c85121_g1_i1 | -1.14 | -6.920 | 7.07E-15 | 8.79E-12 | COL5_ARATH | GO:0003700:transcription factor activity, sequence-specific DNA binding | ATCOL5 |
| c92901_g1 | c92901_g1_i2 | -1.092 | -1.805 | 2.68E-05 | 0.002575167 | BZP61_ARATH | GO:0003700:transcription factor activity, sequence-specific DNA binding |  |
| c84429_g2 | c84429_g2_i1 | -1.073 | -3.409 | 5.79E-07 | 0.000109428 | COL16_ARATH | GO:0003700:transcription factor activity, sequence-specific DNA binding |  |
| c95680_g1 | c95680_g1_i1 | -1.019 | -3.338 | 1.24E-06 | 0.000206043 | FHY3_ARATH | GO:0003700:transcription factor activity, sequence-specific DNA binding |  |
| c82793_g2 | c82793_g2_i6 | -0.996 | -1.296 | 0.000288588 | 0.016939909 | . | GO:0016049:cell growth |  |
| c72312_g2 | c72312_g2_i1 | -0.995 | -1.674 | 0.000135919 | 0.009331861 | . | GO:0007275:multicellular organismal development |  |
| c84429_g1 | c84429_g1_i1 | -0.972 | -4.452 | 4.59E-08 | 1.24E-05 | COL16_ARATH | GO:0003700:transcription factor activity, sequence-specific DNA binding |  |
| c96500_g2 | c96500_g2_i2 | -0.953 | -4.758 | 7.54E-09 | 2.44E-06 | LACS6_ARATH | GO:0007275:multicellular organismal development |  |
| c82793_g2 | c82793_g2_i4 | -0.946 | -3.271 | 1.93E-07 | 4.28E-05 | . | GO:0016049:cell growth |  |
| c84429_g2 | c84429_g2_i3 | -0.917 | -1.318 | 0.00122105 | 0.049447386 | COL16_ARATH | GO:0003700:transcription factor activity, sequence-specific DNA binding |  |
| c96500_g2 | c96500_g2_i1 | -0.903 | -4.316 | 9.69E-08 | 2.35E-05 | LACS6_ARATH | GO:0007275:multicellular organismal development |  |
| c94667_g1 | c94667_g1_i10 | -0.893 | -2.896 | 3.57E-05 | 0.003247307 | RIR2A_ARATH | GO:0007275:multicellular organismal development |  |
| c77870_g1 | c77870_g1_i1 | -0.881 | -5.042 | 8.21E-05 | 0.006309375 | NAC25_ARATH | GO:0003700:transcription factor activity, sequence-specific DNA binding |  |
| c83760_g2 | c83760_g2_i1 | -0.867 | -7.285 | 1.47E-06 | 0.00024067 | COL16_ARATH | GO:0003700:transcription factor activity, sequence-specific DNA binding |  |
| c74691_g1 | c74691_g1_i1 | -0.83 | -3.735 | 5.55E-07 | 0.000105726 | IBH1_ORYSJ | GO:0009826:unidimensional cell growth |  |
| c92467_g2 | c92467_g2_i1 | -0.817 | -6.056 | 8.02E-08 | 2.02E-05 | PCLL_ARATH | GO:0003700:transcription factor activity, sequence-specific DNA binding |  |
| c82793_g2 | c82793_g2_i2 | -0.785 | -3.881 | 5.42E-07 | 0.000103792 | AHP1_ARATH | GO:0016049:cell growth |  |
| c82793_g2 | c82793_g2_i5 | -0.784 | -1.869 | 0.001159125 | 0.047690475 | . | GO:0016049:cell growth |  |
| c87720_g3 | c87720_g3_i2 | -0.784 | -4.393 | 0.000218997 | 0.013658358 | C85A1_SOLLC | GO:0016132:brassinosteroid biosynthetic process |  |
| c95161_g2 | c95161_g2_i3 | -0.762 | -3.548 | 0.000179151 | 0.011676794 | C90B1_ARATH | GO:0016132:brassinosteroid biosynthetic process |  |
| c85522_g2 | c85522_g2_i1 | -0.731 | -2.232 | 0.000923325 | 0.040283689 | . | GO:0003700:transcription factor activity, sequence-specific DNA binding |  |
| c68977_g1 | c68977_g1_i1 | -0.72 | -2.637 | 0.000280544 | 0.016623621 | BH061_ARATH | GO:0003700:transcription factor activity, sequence-specific DNA binding |  |
| c87720_g3 | c87720_g3_i1 | -0.704 | -4.563 | 0.000808138 | 0.036484952 | C85A1_SOLLC | GO:0016132:brassinosteroid biosynthetic process |  |
| c80490_g1 | c80490_g1_i2 | -0.693 | -4.345 | 3.87E-06 | 0.000533198 | WRK65_ARATH | GO:0003700:transcription factor activity, sequence-specific DNA binding |  |
| c95597_g1 | c95597_g1_i1 | -0.69 | -5.461 | 4.15E-05 | 0.003667952 | C90A1_ARATH | GO:0016132:brassinosteroid biosynthetic process |  |
| c68977_g1 | c68977_g1_i2 | -0.675 | -3.471 | 0.000517273 | 0.0261814 | BH061_ARATH | GO:0003700:transcription factor activity, sequence-specific DNA binding |  |
| c94690_g4 | c94690_g4_i2 | -0.657 | -4.161 | 2.96E-05 | 0.002809725 | SPT20_HUMAN | GO:0007275:multicellular organismal development |  |
| c81577_g4 | c81577_g4_i2 | -0.632 | -3.577 | 0.001387497 | 0.054291051 | GAT24_ARATH | GO:0003700:transcription factor activity, sequence-specific DNA binding |  |
| c94690_g4 | c94690_g4_i3 | -0.62 | -4.157 | 0.000144665 | 0.009803349 | SPT20_HUMAN | GO:0007275:multicellular organismal development |  |
| c80364_g1 | c80364_g1_i1 | -0.609 | -2.866 | 0.000821619 | 0.03693627 | CESA6_ARATH | GO:0016049:cell growth |  |
| c95593_g1 | c95593_g1_i1 | -0.608 | -3.580 | 0.000354279 | 0.019684559 | SCL14_ARATH | GO:0003700:transcription factor activity, sequence-specific DNA binding |  |
| c78992_g4 | c78992_g4_i1 | -0.584 | -6.079 | 0.000178149 | 0.011627632 | NFYC1_ARATH | GO:0003700:transcription factor activity, sequence-specific DNA binding | HAP5C3 |
| c85522_g2 | c85522_g2_i2 | -0.576 | -5.474 | 1.56E-05 | 0.001667734 | BH104_ARATH | GO:0003700:transcription factor activity, sequence-specific DNA binding |  |
| c95161_g2 | c95161_g2_i2 | -0.553 | -4.085 | 0.001071708 | 0.045006142 | C90B1_ARATH | GO:0016132:brassinosteroid biosynthetic process |  |
| c92766_g2 | c92766_g2_i1 | -0.537 | -3.840 | 0.000316031 | 0.018103741 | GAT21_ARATH | GO:0009740:gibberellic acid mediated signaling pathway |  |
| c96383_g2 | c96383_g2_i1 | -0.527 | -3.950 | 0.001015556 | 0.043193668 | PHL1_ARATH | GO:0003700:transcription factor activity, sequence-specific DNA binding |  |
| c91463_g1 | c91463_g1_i1 | -0.521 | -5.366 | 6.46E-05 | 0.005229199 | DOF16_ARATH | GO:0003700:transcription factor activity, sequence-specific DNA binding |  |
| c76098_g1 | c76098_g1_i1 | -0.52 | -5.145 | 0.000310193 | 0.017867396 | . | GO:0009740:gibberellic acid mediated signaling pathway |  |
| c94690_g4 | c94690_g4_i1 | -0.506 | -4.432 | 0.00045071 | 0.023581468 | SPT20_HUMAN | GO:0007275:multicellular organismal development |  |
| c77786_g1 | c77786_g1_i1 | -0.503 | -6.294 | 0.000835823 | 0.037422507 | COL3_ARATH | GO:0003700:transcription factor activity, sequence-specific DNA binding |  |
| c84745_g3 | c84745_g3_i1 | -0.492 | -4.365 | 0.000453215 | 0.023672952 | ILR3_ARATH | GO:0003700:transcription factor activity, sequence-specific DNA binding |  |
| c96122_g2 | c96122_g2_i2 | 0.481 | -4.782 | 0.000273822 | 0.016312842 | NAC86_ARATH | GO:0003700:transcription factor activity, sequence-specific DNA binding |  |
| c87551_g2 | c87551_g2_i1 | 0.527 | -3.386 | 0.001381023 | 0.054105401 | NAC78_ARATH | GO:0003700:transcription factor activity, sequence-specific DNA binding |  |
| c86454_g4 | c86454_g4_i1 | 0.532 | -3.678 | 0.000983738 | 0.042174756 | SCL1_ARATH | GO:0003700:transcription factor activity, sequence-specific DNA binding |  |
| c84242_g1 | c84242_g1_i1 | 0.587 | -4.159 | 7.00E-05 | 0.005565697 | KAN2_ARATH | GO:0003700:transcription factor activity, sequence-specific DNA binding |  |
| c79824_g2 | c79824_g2_i1 | 0.592 | -3.778 | 0.000135763 | 0.009328 | AHP1_ARATH | GO:0016049:cell growth |  |
| c85059_g3 | c85059_g3_i1 | 0.61 | -4.304 | 0.000735385 | 0.033978801 | ZWIP2_ARATH | GO:0003700:transcription factor activity, sequence-specific DNA binding |  |
| c93184_g1 | c93184_g1_i1 | 0.611 | -4.704 | 1.27E-05 | 0.001415544 | KATAM_ARATH | GO:0009826:unidimensional cell growth |  |
| c87551_g2 | c87551_g2_i3 | 0.614 | -2.824 | 0.000678802 | 0.032040734 | NAC78_ARATH | GO:0003700:transcription factor activity, sequence-specific DNA binding |  |
| c83494_g1 | c83494_g1_i1 | 0.636 | -2.800 | 0.000949463 | 0.041127828 | MYB44_ARATH | GO:0003700:transcription factor activity, sequence-specific DNA binding |  |
| c82913_g1 | c82913_g1_i1 | 0.652 | -7.717 | 1.09E-05 | 0.001259723 | RA212_ARATH | GO:0003700:transcription factor activity, sequence-specific DNA binding |  |
| c93573_g3 | c93573_g3_i1 | 0.665 | -5.111 | 0.000115588 | 0.008212263 | GMD2_ARATH | GO:0009826:unidimensional cell growth |  |
| c91032_g1 | c91032_g1_i1 | 0.67 | -6.364 | 0.0001556 | 0.010412862 | EF118_ARATH | GO:0003700:transcription factor activity, sequence-specific DNA binding |  |
| c96979_g6 | c96979_g6_i2 | 0.671 | -3.721 | 0.000246754 | 0.015019511 | ANT_ARATH | GO:0003700:transcription factor activity, sequence-specific DNA binding | TaQ |
| c91032_g1 | c91032_g1_i2 | 0.697 | -6.031 | 1.04E-05 | 0.00121331 | EF118_ARATH | GO:0003700:transcription factor activity, sequence-specific DNA binding |  |
| 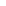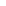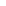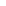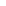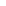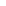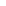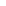   | c79540_g1 | | --- | | c79540_g1_i4 | 0.697 | -2.855 | 0.000612247 | 0.029630687 | MY113_ARATH | GO:0003700:transcription factor activity, sequence-specific DNA binding |  |
| c85724_g1 | c85724_g1_i1 | 0.698 | -4.849 | 3.34E-05 | 0.003085236 | AI5L5_ARATH | GO:0003700:transcription factor activity, sequence-specific DNA binding | ABF2 |
| c92052_g1 | c92052_g1_i3 | 0.701 | -3.172 | 0.000298677 | 0.017406988 | ASIL2_ARATH | GO:0003700:transcription factor activity, sequence-specific DNA binding |  |
| c98058_g2 | c98058_g2_i1 | 0.705 | -3.549 | 0.000903137 | 0.039633108 | BH030_ARATH | GO:0003700:transcription factor activity, sequence-specific DNA binding |  |
| c98058_g2 | c98058_g2_i2 | 0.716 | -2.576 | 0.000982509 | 0.042131648 | . | GO:0003700:transcription factor activity, sequence-specific DNA binding |  |
| c87551_g2 | c87551_g2_i5 | 0.734 | -3.310 | 2.74E-05 | 0.002630348 | NAC78_ARATH | GO:0003700:transcription factor activity, sequence-specific DNA binding |  |
| c90658_g1 | *c90658_g1_i5 | 0.746 | -2.963 | 5.25E-05 | 0.004452248 | NLP7_ARATH | GO:0003700:transcription factor activity, sequence-specific DNA binding |  |
| c90658_g1 | *c90658_g1_i5 | 0.746 | -2.963 | 5.25E-05 | 0.004452248 | NLP9_ARATH | GO:0003700:transcription factor activity, sequence-specific DNA binding |  |
| c83530_g1 | c83530_g1_i2 | 0.752 | -3.232 | 0.000191064 | 0.012324921 | WRK70_ARATH | GO:0003700:transcription factor activity, sequence-specific DNA binding |  |
| c93556_g1 | c93556_g1_i2 | 0.773 | -2.054 | 0.001359145 | 0.053506074 | ARR11_ARATH | GO:0003700:transcription factor activity, sequence-specific DNA binding |  |
| c86516_g1 | c86516_g1_i1 | 0.784 | -5.507 | 1.28E-07 | 3.00E-05 | BBX24_ARATH | GO:0003700:transcription factor activity, sequence-specific DNA binding |  |
| c93310_g3 | c93310_g3_i1 | 0.812 | -6.050 | 5.11E-05 | 0.004354167 | SPL5_ARATH | GO:0003700:transcription factor activity, sequence-specific DNA binding |  |
| c94725_g2 | c94725_g2_i1 | 0.817 | -2.724 | 0.000172796 | 0.011321489 | NAC7_ARATH | GO:0003700:transcription factor activity, sequence-specific DNA binding |  |
| c83991_g1 | c83991_g1_i1 | 0.819 | -2.487 | 0.00021033 | 0.013257989 | BZP61_ARATH | GO:0003700:transcription factor activity, sequence-specific DNA binding |  |
| c80101_g1 | c80101_g1_i1 | 0.826 | -2.753 | 9.86E-05 | 0.00725468 | COBL4_ARATH | GO:0016049:cell growth |  |
| c89989_g1 | c89989_g1_i1 | 0.834 | -3.274 | 3.33E-05 | 0.003072855 | DOF35_ARATH | GO:0003700:transcription factor activity, sequence-specific DNA binding |  |
| c88138_g1 | c88138_g1_i1 | 0.845 | -2.269 | 0.000281656 | 0.016642882 | NLP5_ARATH | GO:0003700:transcription factor activity, sequence-specific DNA binding |  |
| c86280_g3 | c86280_g3_i1 | 0.846 | -2.745 | 2.40E-05 | 0.002356637 |  | GO:0003700:transcription factor activity, sequence-specific DNA binding | CHE |
| c93832_g4 | c93832_g4_i2 | 0.85 | -1.643 | 0.000709947 | 0.033063766 | BH074_ARATH | GO:0003700:transcription factor activity, sequence-specific DNA binding | CIB1 |
| c92695_g1 | c92695_g1_i1 | 0.853 | -7.215 | 0.000103004 | 0.007508501 | WAT1_ARATH | GO:0009826:unidimensional cell growth |  |
| c96544_g4 | c96544_g4_i1 | 0.856 | -3.408 | 6.41E-06 | 0.000813005 | ULT1_ARATH | GO:0007275:multicellular organismal development |  |
| c83856_g1 | *c83856_g1_i2 | 0.863 | -3.078 | 1.67E-05 | 0.001769962 | GID1B_ARATH | GO:0009739:response to gibberellin |  |
| c83856_g1 | *c83856_g1_i2 | 0.863 | -3.078 | 1.67E-05 | 0.001769962 | GID1B_ARATH | GO:0009740:gibberellic acid mediated signaling pathway |  |
| c96979_g6 | c96979_g6_i1 | 0.865 | -3.461 | 4.35E-05 | 0.003801832 | ANT_ARATH | GO:0003700:transcription factor activity, sequence-specific DNA binding | LOC_Os06g10880.1 |
| c99655_g5 | c99655_g5_i9 | 0.866 | -2.316 | 0.000156787 | 0.010462409 | NLP7_ARATH | GO:0003700:transcription factor activity, sequence-specific DNA binding |  |
| c93556_g1 | *c93556_g1_i1 | 0.866 | -3.672 | 8.88E-06 | 0.001065753 | ARR11_ARATH | GO:0003700:transcription factor activity, sequence-specific DNA binding |  |
| c93556_g1 | *c93556_g1_i1 | 0.866 | -3.672 | 8.88E-06 | 0.001065753 | ARR11_ARATH | GO:0003700:transcription factor activity, sequence-specific DNA binding |  |
| c77902_g3 | c77902_g3_i1 | 0.874 | -3.289 | 0.000309791 | 0.017849707 | PSK6_ARATH | GO:0007275:multicellular organismal development |  |
| c97270_g1 | c97270_g1_i2 | 0.878 | -3.553 | 4.52E-08 | 1.23E-05 | . | GO:0003700:transcription factor activity, sequence-specific DNA binding |  |
| c96879_g4 | c96879_g4_i1 | 0.889 | -4.895 | 5.20E-12 | 3.46E-09 | RVE6_ARATH | GO:0003700:transcription factor activity, sequence-specific DNA binding | OsCCA1 |
| c83742_g1 | c83742_g1_i1 | 0.917 | -4.453 | 4.84E-07 | 9.45E-05 | AGL8_SOLTU | GO:0003700:transcription factor activity, sequence-specific DNA binding | OsMADS18 |
| c99991_g4 | c99991_g4_i1 | 0.927 | -2.078 | 0.000125793 | 0.008751867 | BH051_ARATH | GO:0003700:transcription factor activity, sequence-specific DNA binding |  |
| c79829_g1 | c79829_g1_i2 | 0.967 | -1.505 | 0.000202488 | 0.012865483 | COL1_ARATH | GO:0003700:transcription factor activity, sequence-specific DNA binding |  |
| c93832_g4 | c93832_g4_i1 | 0.985 | -2.419 | 2.45E-06 | 0.000367673 | BH074_ARATH | GO:0003700:transcription factor activity, sequence-specific DNA binding | CIB1 |
| c90612_g1 | c90612_g1_i2 | 0.986 | -3.833 | 3.50E-07 | 7.16E-05 | ERF61_ARATH | GO:0003700:transcription factor activity, sequence-specific DNA binding |  |
| c73918_g2 | c73918_g2_i1 | 0.996 | -1.045 | 0.000582511 | 0.028544691 | MYB46_ARATH | GO:0003700:transcription factor activity, sequence-specific DNA binding |  |
| c93310_g3 | c93310_g3_i2 | 1.01 | -3.801 | 9.37E-06 | 0.001115884 | SPL5_ARATH | GO:0003700:transcription factor activity, sequence-specific DNA binding |  |
| c78879_g2 | c78879_g2_i1 | 1.011 | -3.135 | 0.000249129 | 0.015129723 | . | GO:0003700:transcription factor activity, sequence-specific DNA binding |  |
| c98245_g3 | c98245_g3_i1 | 1.016 | -2.739 | 6.27E-06 | 0.000798697 | SRO2_ARATH | GO:0007275:multicellular organismal development |  |
| c90612_g1 | c90612_g1_i1 | 1.025 | -3.943 | 6.47E-07 | 0.000119067 | ERF61_ARATH | GO:0003700:transcription factor activity, sequence-specific DNA binding |  |
| c82641_g2 | c82641_g2_i1 | 1.031 | -2.238 | 4.52E-05 | 0.003919205 | BH110_ARATH | GO:0003700:transcription factor activity, sequence-specific DNA binding |  |
| c99655_g5 | c99655_g5_i10 | 1.032 | -2.551 | 1.41E-05 | 0.001536969 | NLP7_ARATH | GO:0003700:transcription factor activity, sequence-specific DNA binding |  |
| c91118_g5 | c91118_g5_i1 | 1.047 | -1.188 | 0.000248602 | 0.015107544 | BH032_ARATH | GO:0003700:transcription factor activity, sequence-specific DNA binding |  |
| c97270_g1 | c97270_g1_i5 | 1.048 | -0.700 | 0.001385729 | 0.054244488 | NLP7_ARATH | GO:0003700:transcription factor activity, sequence-specific DNA binding |  |
| c86797_g4 | *c86797_g4_i1 | 1.082 | -1.789 | 0.000450012 | 0.023571238 | . | GO:0003700:transcription factor activity, sequence-specific DNA binding |  |
| c86797_g4 | *c86797_g4_i1 | 1.082 | -1.789 | 0.000450012 | 0.023571238 | . | GO:0009739:response to gibberellin |  |
| c97280_g1 | c97280_g1_i3 | 1.087 | -2.623 | 5.97E-05 | 0.004935201 | MYB5_ARATH | GO:0003700:transcription factor activity, sequence-specific DNA binding |  |
| c77389_g1 | c77389_g1_i1 | 1.102 | -1.482 | 0.000609817 | 0.029551185 | CRF6_ARATH | GO:0003700:transcription factor activity, sequence-specific DNA binding |  |
| c89086_g6 | *c89086_g6_i1 | 1.107 | -5.416 | 6.97E-11 | 3.81E-08 | MYB28_ARATH | GO:0003700:transcription factor activity, sequence-specific DNA binding |  |
| c89086_g6 | *c89086_g6_i1 | 1.107 | -5.416 | 6.97E-11 | 3.81E-08 | MYB28_ARATH | GO:0009739:response to gibberellin |  |
| c74422_g1 | c74422_g1_i1 | 1.121 | -4.001 | 4.13E-12 | 2.84E-09 | . | GO:0007275:multicellular organismal development |  |
| c95664_g3 | c95664_g3_i1 | 1.142 | -2.162 | 1.39E-05 | 0.001523899 | NAC8_ARATH | GO:0003700:transcription factor activity, sequence-specific DNA binding |  |
| c74782_g1 | c74782_g1_i1 | 1.18 | -2.472 | 2.87E-08 | 8.21E-06 | WIN1_ARATH | GO:0003700:transcription factor activity, sequence-specific DNA binding |  |
| c92684_g3 | c92684_g3_i2 | 1.188 | -2.755 | 8.30E-09 | 2.67E-06 | C3H20_ARATH | GO:0003700:transcription factor activity, sequence-specific DNA binding |  |
| c100314_g1 | c100314_g1_i3 | 1.199 | -4.567 | 9.61E-14 | 9.50E-11 | NLP7_ARATH | GO:0003700:transcription factor activity, sequence-specific DNA binding |  |
| c84971_g2 | c84971_g2_i1 | 1.228 | -4.121 | 1.39E-07 | 3.22E-05 | IAA14_ARATH | GO:0003700:transcription factor activity, sequence-specific DNA binding |  |
| c83405_g2 | c83405_g2_i1 | 1.231 | -1.978 | 5.42E-07 | 0.000103792 | NAC8_ARATH | GO:0003700:transcription factor activity, sequence-specific DNA binding |  |
| c92684_g3 | c92684_g3_i1 | 1.274 | -3.114 | 7.05E-09 | 2.30E-06 | C3H49_ARATH | GO:0003700:transcription factor activity, sequence-specific DNA binding |  |
| c58787_g1 | c58787_g1_i1 | 1.281 | -1.618 | 0.000748051 | 0.034420041 | RADL1_ARATH | GO:0003700:transcription factor activity, sequence-specific DNA binding |  |
| c92984_g1 | c92984_g1_i2 | 1.281 | -1.954 | 0.001210845 | 0.049161354 | KO1_ARATH | GO:0009740:gibberellic acid mediated signaling pathway |  |
| c86010_g1 | c86010_g1_i1 | 1.297 | -4.487 | 3.59E-12 | 2.52E-09 | SOC1_ARATH | GO:0003700:transcription factor activity, sequence-specific DNA binding | AGL19 |
| c86010_g1 | c86010_g1_i1 | 1.297 | -4.487 | 3.59E-12 | 2.52E-09 | SOC1_ARATH | GO:0009739:response to gibberellin | SOC1 |
| c92984_g1 | c92984_g1_i1 | 1.302 | -2.772 | 2.86E-05 | 0.002721058 | KO1_ARATH | GO:0009740:gibberellic acid mediated signaling pathway |  |
| c85645_g3 | c85645_g3_i2 | 1.322 | -1.568 | 6.54E-06 | 0.000826542 | G2OX1_PEA | GO:0009739:response to gibberellin |  |
| c86010_g1 | *c86010_g1_i3 | 1.34 | -4.510 | 2.89E-12 | 2.11E-09 | SOC1_ARATH | GO:0003700:transcription factor activity, sequence-specific DNA binding | SOC1 |
| c86010_g1 | *c86010_g1_i3 | 1.34 | -4.510 | 2.89E-12 | 2.11E-09 | SOC1_ARATH | GO:0003700:transcription factor activity, sequence-specific DNA binding | SOC1 |
| c86010_g1 | *c86010_g1_i3 | 1.34 | -4.510 | 2.89E-12 | 2.11E-09 | SOC1_ARATH | GO:0009739:response to gibberellin | SOC1 |
| c94250_g2 | c94250_g2_i1 | 1.341 | -3.002 | 1.51E-07 | 3.43E-05 | MYB12_ARATH | GO:0003700:transcription factor activity, sequence-specific DNA binding |  |
| c70648_g1 | c70648_g1_i1 | 1.371 | -0.353 | 0.000501166 | 0.025607753 | G3OX_PEA | GO:0009739:response to gibberellin |  |
| c92984_g1 | c92984_g1_i3 | 1.422 | -1.397 | 1.12E-05 | 0.001282792 | KO1_ARATH | GO:0009740:gibberellic acid mediated signaling pathway |  |
| c86010_g1 | *c86010_g1_i2 | 1.429 | -4.165 | 7.43E-14 | 7.58E-11 | SOC1_ARATH | GO:0003700:transcription factor activity, sequence-specific DNA binding | AGL19 |
| c86010_g1 | *c86010_g1_i2 | 1.429 | -4.165 | 7.43E-14 | 7.58E-11 | SOC1_ARATH | GO:0009739:response to gibberellin | SOC1 |
| c100314_g1 | c100314_g1_i2 | 1.497 | -0.185 | 0.000446392 | 0.023460143 | . | GO:0003700:transcription factor activity, sequence-specific DNA binding |  |
| c84014_g1 | c84014_g1_i3 | 1.503 | -1.355 | 4.67E-05 | 0.00403568 | IAA14_ARATH | GO:0003700:transcription factor activity, sequence-specific DNA binding |  |
| c81046_g1 | c81046_g1_i1 | 1.506 | -6.289 | 5.86E-05 | 0.004863686 | MYB5_ARATH | GO:0003700:transcription factor activity, sequence-specific DNA binding |  |
| c81260_g1 | c81260_g1_i2 | 1.523 | -4.413 | 4.36E-12 | 2.96E-09 | SPL6_ARATH | GO:0003700:transcription factor activity, sequence-specific DNA binding | SPL |
| c55095_g1 | c55095_g1_i1 | 1.566 | -0.166 | 0.000564186 | 0.027916264 | ZFP6_ARATH | GO:0009740:gibberellic acid mediated signaling pathway |  |
| c81260_g1 | c81260_g1_i1 | 1.575 | -3.998 | 1.49E-13 | 1.40E-10 | SPL6_ARATH | GO:0003700:transcription factor activity, sequence-specific DNA binding |  |
| c94167_g3 | c94167_g3_i1 | 1.578 | -4.776 | 7.82E-07 | 0.000138831 | ARFE_ARATH | GO:0003700:transcription factor activity, sequence-specific DNA binding |  |
| c72664_g1 | c72664_g1_i3 | 1.588 | -1.223 | 6.28E-08 | 1.64E-05 | NLP6_ARATH | GO:0003700:transcription factor activity, sequence-specific DNA binding |  |
| c79733_g1 | c79733_g1_i1 | 1.659 | 0.373 | 0.000425157 | 0.022571525 | ACAP1_ARATH | GO:0009826:unidimensional cell growth |  |
| c84660_g1 | c84660_g1_i1 | 1.681 | -1.967 | 0.000119757 | 0.008439524 | MYB3_ARATH | GO:0003700:transcription factor activity, sequence-specific DNA binding |  |
| c88221_g2 | c88221_g2_i3 | 1.686 | -6.222 | 1.22E-09 | 4.92E-07 | KNAT6_ARATH | GO:0003700:transcription factor activity, sequence-specific DNA binding |  |
| c84014_g1 | c84014_g1_i2 | 1.763 | -1.328 | 1.29E-06 | 0.00021463 | IAA14_ARATH | GO:0003700:transcription factor activity, sequence-specific DNA binding |  |
| c61782_g1 | c61782_g1_i1 | 1.872 | -0.764 | 0.000314968 | 0.018064843 | RAV1_ARATH | GO:0003700:transcription factor activity, sequence-specific DNA binding | RAV1 |
| c84014_g1 | c84014_g1_i1 | 1.931 | -1.556 | 5.48E-08 | 1.45E-05 | IAA14_ARATH | GO:0003700:transcription factor activity, sequence-specific DNA binding |  |
| c86851_g1 | c86851_g1_i5 | 1.945 | -1.381 | 0.000146643 | 0.009912307 | BH047_ARATH | GO:0003700:transcription factor activity, sequence-specific DNA binding |  |
| c85645_g3 | c85645_g3_i3 | 1.959 | -0.157 | 2.48E-06 | 0.00037129 | G2OX1_PEA | GO:0009739:response to gibberellin |  |
| c86504_g1 | c86504_g1_i2 | 2.187 | 0.646 | 4.98E-05 | 0.004258032 | HMGB7_ARATH | GO:0003700:transcription factor activity, sequence-specific DNA binding |  |
| c77826_g1 | c77826_g1_i1 | 2.19 | -1.374 | 3.18E-12 | 2.27E-09 | . | GO:0003700:transcription factor activity, sequence-specific DNA binding |  |
| c108905_g1 | c108905_g1_i1 | 2.342 | -0.026 | 1.90E-05 | 0.001965926 | . | GO:0003700:transcription factor activity, sequence-specific DNA binding |  |
| c86851_g1 | c86851_g1_i6 | 2.687 | -1.151 | 1.32E-05 | 0.001454504 | BH047_ARATH | GO:0003700:transcription factor activity, sequence-specific DNA binding |  |
| c94962_g1 | c94962_g1_i1 | 2.7 | -0.919 | 3.82E-11 | 2.20E-08 | NLP7_ARATH | GO:0003700:transcription factor activity, sequence-specific DNA binding |  |
| c96427_g2 | c96427_g2_i2 | 2.782 | 0.135 | 4.92E-08 | 1.32E-05 | FL1_TOBAC | GO:0007275:multicellular organismal development | LFY |
| c83775_g1 | c83775_g1_i2 | 2.796 | 0.326 | 2.49E-05 | 0.002427502 | KNAT6_ARATH | GO:0003700:transcription factor activity, sequence-specific DNA binding |  |
| c91057_g4 | c91057_g4_i2 | 2.838 | 0.725 | 1.01E-05 | 0.001177617 | ERF43_ARATH | GO:0003700:transcription factor activity, sequence-specific DNA binding |  |
| c82887_g1 | c82887_g1_i1 | 2.864 | 1.421 | 0.000987842 | 0.042289508 | MY108_ARATH | GO:0003700:transcription factor activity, sequence-specific DNA binding |  |
| c91057_g4 | *c91057_g4_i4 | 3.079 | -0.439 | 2.85E-14 | 3.24E-11 | ERF43_ARATH | GO:0003700:transcription factor activity, sequence-specific DNA binding |  |
| c91057_g4 | *c91057_g4_i4 | 3.079 | -0.439 | 2.85E-14 | 3.24E-11 | DREB3_ARATH | GO:0003700:transcription factor activity, sequence-specific DNA binding |  |
| c86851_g1 | c86851_g1_i2 | 3.103 | 1.115 | 0.001287538 | 0.051408155 | BH047_ARATH | GO:0003700:transcription factor activity, sequence-specific DNA binding |  |
| c86851_g1 | c86851_g1_i3 | 3.122 | -0.114 | 8.03E-05 | 0.006216883 | BH047_ARATH | GO:0003700:transcription factor activity, sequence-specific DNA binding |  |
| c91308_g1 | c91308_g1_i1 | 3.135 | -0.345 | 2.15E-10 | 1.04E-07 | BH149_ARATH | GO:0003700:transcription factor activity, sequence-specific DNA binding |  |
| c86851_g1 | c86851_g1_i4 | 3.23 | 0.237 | 0.000512573 | 0.026013627 | . | GO:0003700:transcription factor activity, sequence-specific DNA binding |  |
| c77694_g2 | c77694_g2_i2 | 3.393 | 1.479 | 6.96E-05 | 0.005545488 | WAK2_ARATH | GO:0009826:unidimensional cell growth |  |
| c81830_g2 | c81830_g2_i1 | 3.453 | -0.061 | 3.06E-11 | 1.78E-08 | AGL9_PETHY | GO:0003700:transcription factor activity, sequence-specific DNA binding | AP1 |
| c92021_g1 | c92021_g1_i2 | 3.488 | -2.504 | 4.41E-27 | 1.80E-23 | AGL8_SOLTU | GO:0003700:transcription factor activity, sequence-specific DNA binding | AP1 |
| c91057_g4 | c91057_g4_i3 | 3.554 | -2.803 | 3.79E-44 | 4.18E-40 | DREB3_ARATH | GO:0003700:transcription factor activity, sequence-specific DNA binding |  |
| c91057_g4 | c91057_g4_i1 | 3.614 | -1.071 | 6.22E-22 | 1.58E-18 | ERF43_ARATH | GO:0003700:transcription factor activity, sequence-specific DNA binding |  |
| c80136_g1 | c80136_g1_i1 | 3.869 | -3.057 | 1.00E-41 | 1.05E-37 | MADS6_ORYSJ | GO:0003700:transcription factor activity, sequence-specific DNA binding | AP1 |
| c92021_g1 | c92021_g1_i1 | 4.042 | -2.955 | 3.74E-32 | 2.06E-28 | AGL8_SOLTU | GO:0003700:transcription factor activity, sequence-specific DNA binding | AP1 |
| c86504_g1 | c86504_g1_i4 | 4.171 | 1.615 | 1.11E-05 | 0.001273193 | HMGB7_ARATH | GO:0003700:transcription factor activity, sequence-specific DNA binding |  |
| c88116_g1 | c88116_g1_i1 | 4.733 | -0.318 | 1.07E-17 | 1.85E-14 | AGL8_SOLTU | GO:0003700:transcription factor activity, sequence-specific DNA binding | FUL |
| c91643_g2 | c91643_g2_i1 | 5.172 | -1.915 | 3.24E-30 | 1.52E-26 | RSP4_CAEEL | GO:0007275:multicellular organismal development |  |
| c88116_g8 | c88116_g8_i1 | 5.774 | 2.357 | 0.000766899 | 0.035029826 | AGL8_SOLTU | GO:0003700:transcription factor activity, sequence-specific DNA binding | AP1 |
| c90323_g1 | c90323_g1_i4 | 5.774 | 2.370 | 0.000332893 | 0.018790296 | MADS6_ORYSJ | GO:0003700:transcription factor activity, sequence-specific DNA binding | FUL |
| c84088_g2 | c84088_g2_i2 | 8.075 | 0.034 | 8.36E-17 | 1.30E-13 | . | GO:0048510:regulation of timing of transition from vegetative to reproductive phase | Hd3a |
| c84088_g2 | c84088_g2_i4 | 8.709 | -0.601 | 1.22E-23 | 3.65E-20 | . | GO:0048510:regulation of timing of transition from vegetative to reproductive phase | FT |
| c87372_g1 | *c87372_g1_i7 | 8.845 | -0.945 | 1.18E-07 | 2.80E-05 | ORG3_ARATH | GO:0003700:transcription factor activity, sequence-specific DNA binding |  |
| c87372_g1 | *c87372_g1_i7 | 8.845 | -0.945 | 1.18E-07 | 2.80E-05 | BH100_ARATH | GO:0003700:transcription factor activity, sequence-specific DNA binding |  |
| c87372_g1 | c87372_g1_i6 | 8.896 | -0.993 | 5.64E-08 | 1.49E-05 | ORG2_ARATH | GO:0003700:transcription factor activity, sequence-specific DNA binding |  |
| c87372_g1 | *c87372_g1_i3 | 8.957 | -1.059 | 8.66E-08 | 2.14E-05 | BH100_ARATH | GO:0003700:transcription factor activity, sequence-specific DNA binding |  |
| c87372_g1 | *c87372_g1_i3 | 8.957 | -1.059 | 8.66E-08 | 2.14E-05 | ORG3_ARATH | GO:0003700:transcription factor activity, sequence-specific DNA binding |  |
| c87372_g1 | c87372_g1_i2 | 9.288 | -1.395 | 3.47E-08 | 9.74E-06 | ORG2_ARATH | GO:0003700:transcription factor activity, sequence-specific DNA binding |  |
| c87372_g1 | c87372_g1_i4 | 9.288 | -1.395 | 3.52E-08 | 9.82E-06 | ORG2_ARATH | GO:0003700:transcription factor activity, sequence-specific DNA binding |  |
| c87372_g1 | *c87372_g1_i1 | 9.307 | -1.411 | 2.07E-08 | 6.10E-06 | ORG3_ARATH | GO:0003700:transcription factor activity, sequence-specific DNA binding |  |
| c87372_g1 | *c87372_g1_i1 | 9.307 | -1.411 | 2.07E-08 | 6.10E-06 | BH100_ARATH | GO:0003700:transcription factor activity, sequence-specific DNA binding |  |
| c84088_g2 | c84088_g2_i6 | 9.454 | -3.688 | 1.42E-77 | 3.33E-73 | . | GO:0048510:regulation of timing of transition from vegetative to reproductive phase | FT |
| c87372_g1 | c87372_g1_i5 | 10.263 | -2.378 | 1.63E-09 | 6.34E-07 | ORG2_ARATH | GO:0003700:transcription factor activity, sequence-specific DNA binding |  |
| c84088_g2 | c84088_g2_i1 | 10.777 | -5.004 | 7.01E-128 | 6.59E-123 | HD3A_ORYSJ | GO:0048510:regulation of timing of transition from vegetative to reproductive phase | FT |
| c84088_g2 | c84088_g2_i5 | 11.782 | -3.702 | 2.21E-104 | 1.04E-99 | HD3A_ORYSJ | GO:0048510:regulation of timing of transition from vegetative to reproductive phase | OsFTL3 |
| c84088_g2 | c84088_g2_i3 | 13.059 | -4.993 | 3.04E-128 | 5.70E-123 | HD3A_ORYSJ | GO:0048510:regulation of timing of transition from vegetative to reproductive phase | OsFTL3 |
