# Supplementary figures and images for: Overexpression of blueberry FLOWERING LOCUS T is associated with changes in the expression of phytohormone-related genes in blueberry plants
Source: Hortic Res. 2016 Oct 26;3:16053–. doi: 10.1038/hortres.2016.53 (PMC5080838; doi:10.1038/hortres.2016.53)

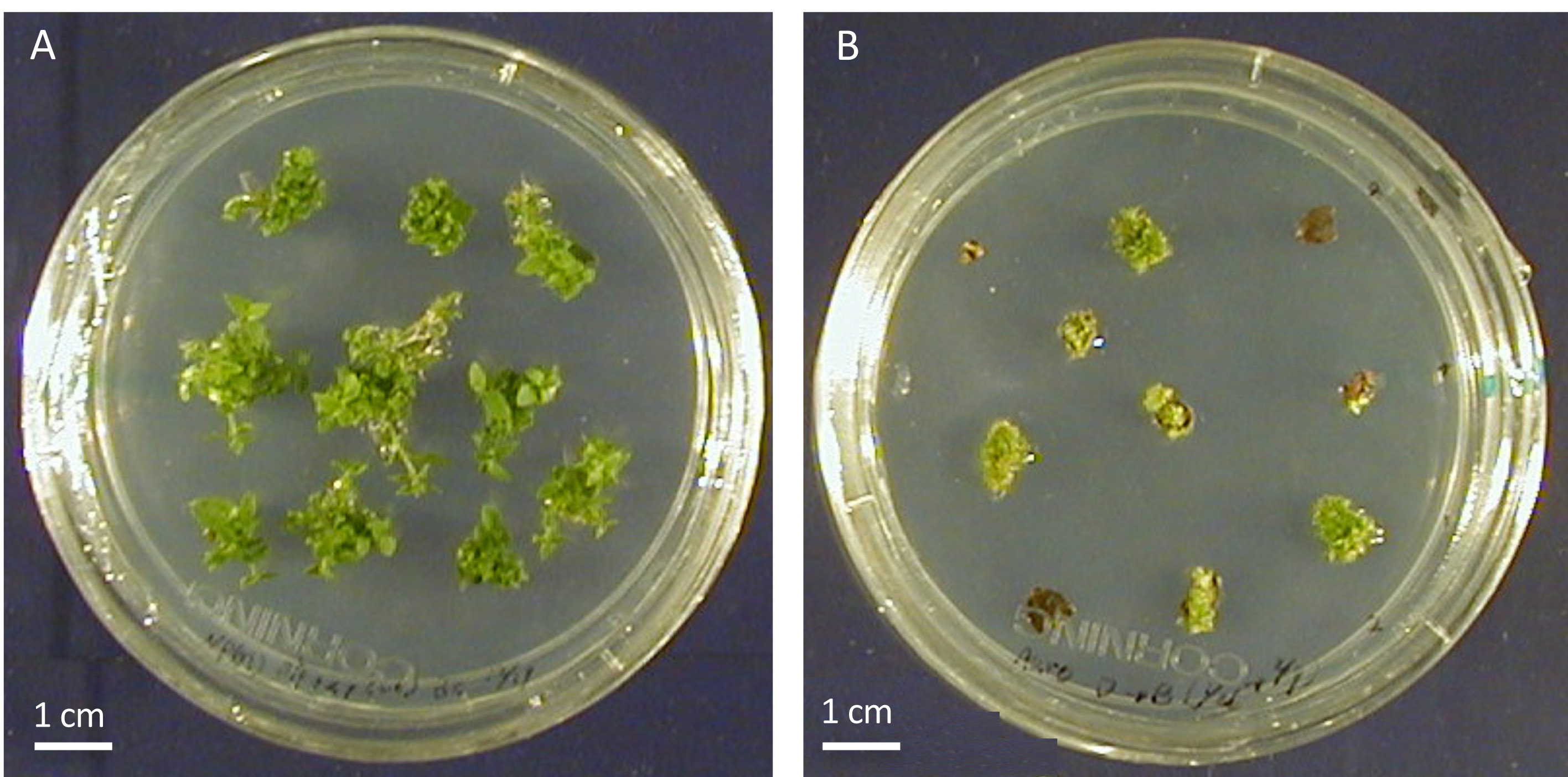

Supplement: Supplementary Figure S1 [file hortres201653-s5.jpg]

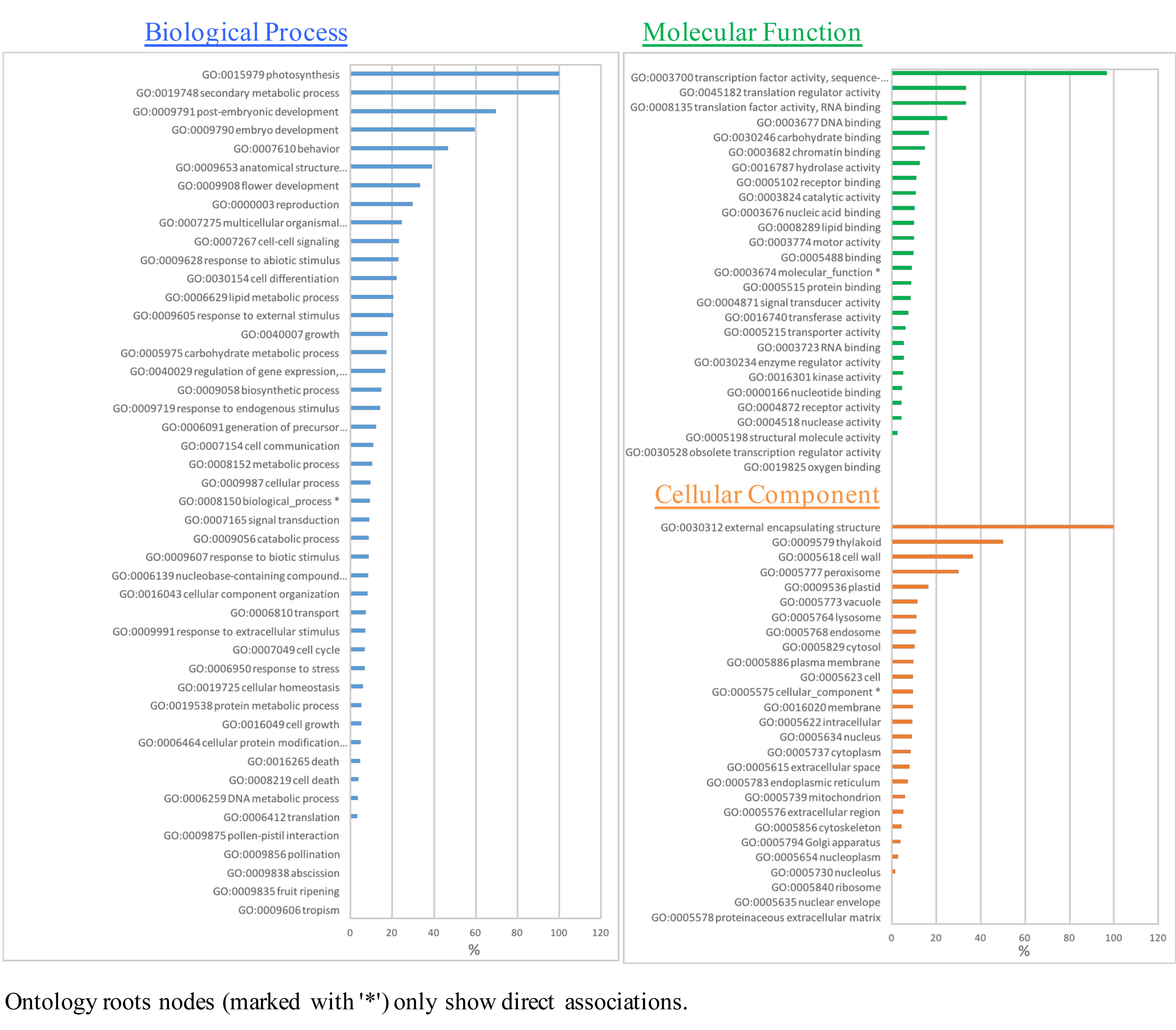

Supplement: Supplementary Figure S2 [file hortres201653-s6.jpg]

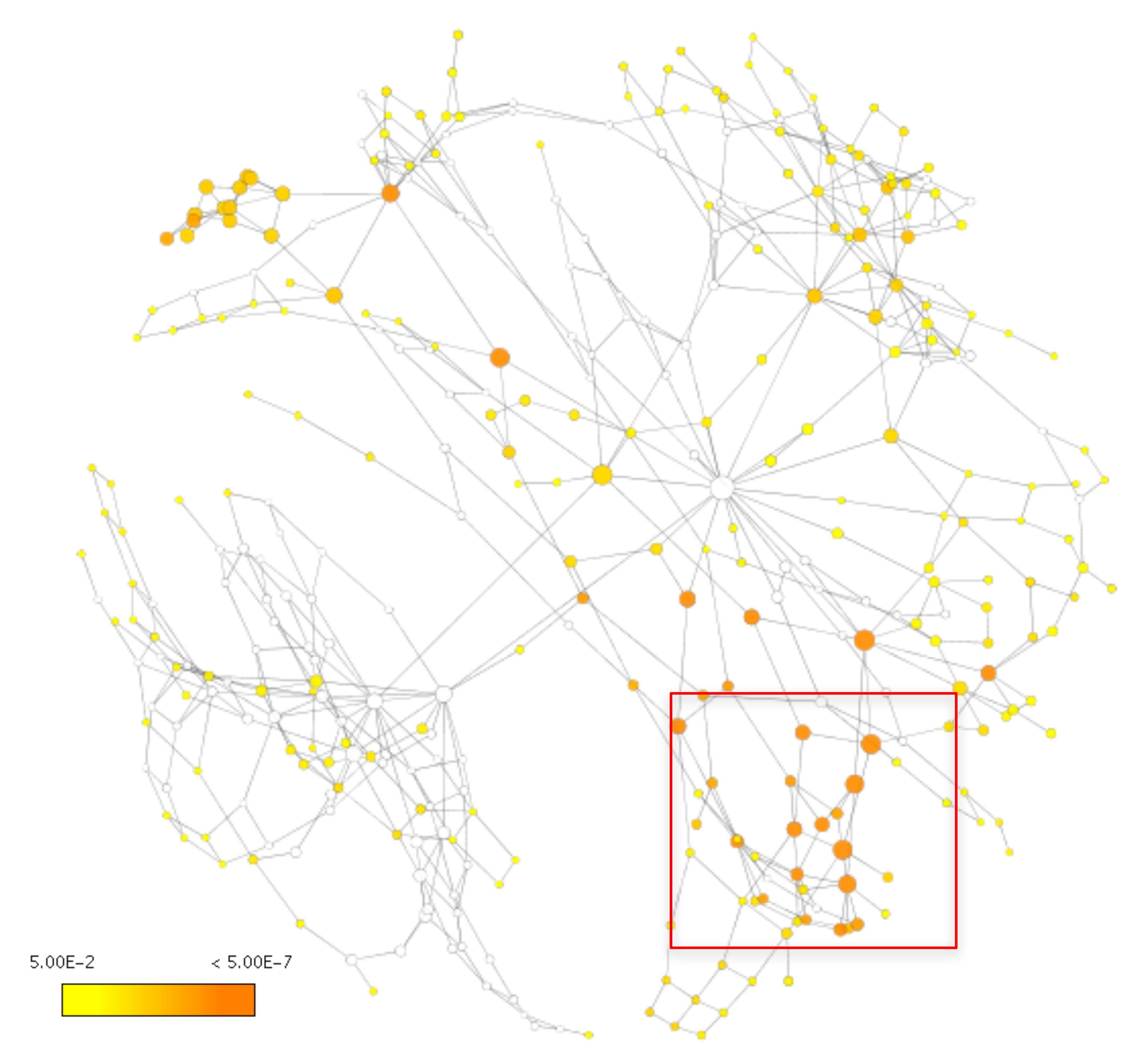

Supplement: Supplementary Figure S3 [file hortres201653-s7.jpg]

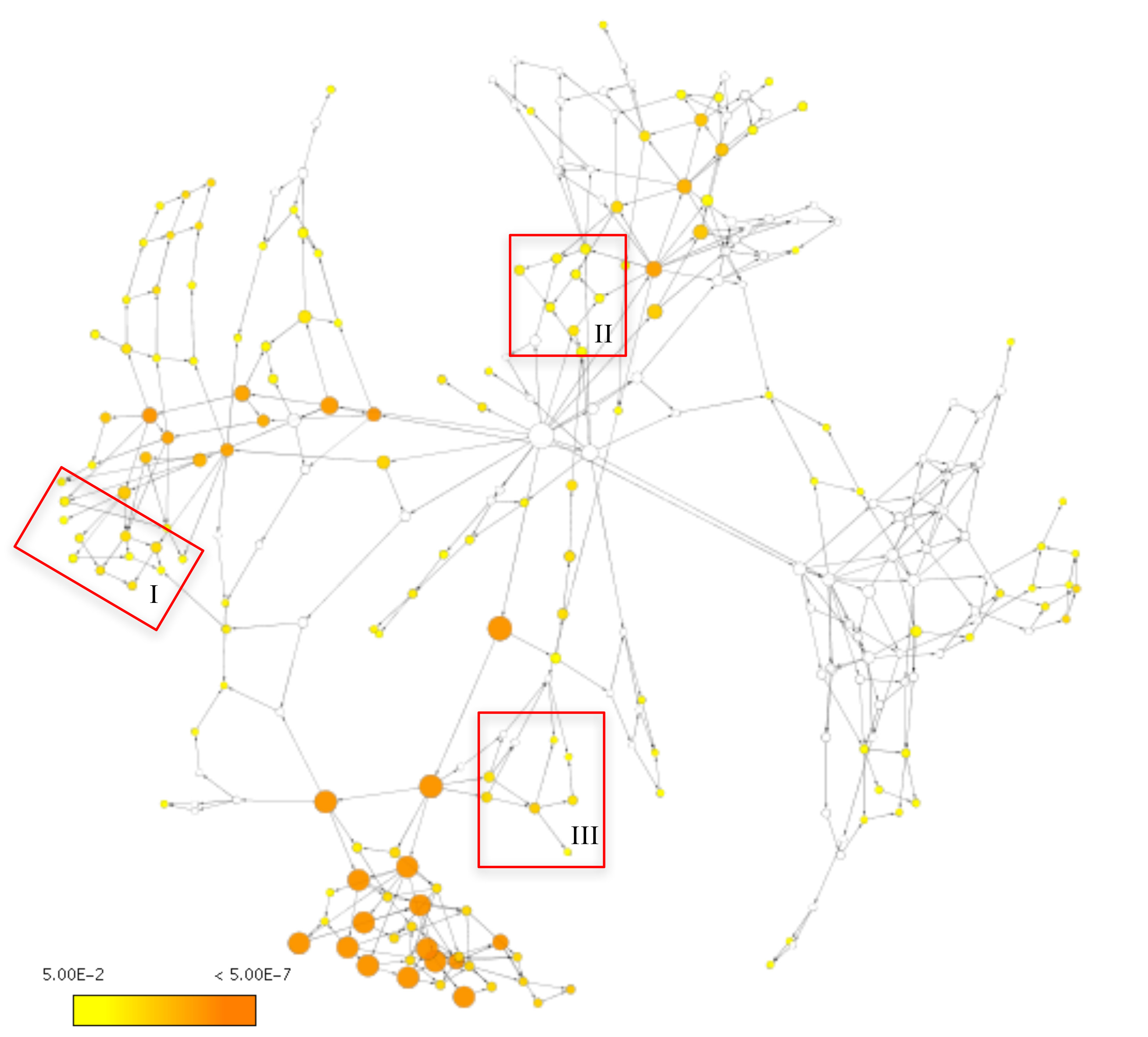

Supplement: Supplementary Figure S4 [file hortres201653-s8.jpg]

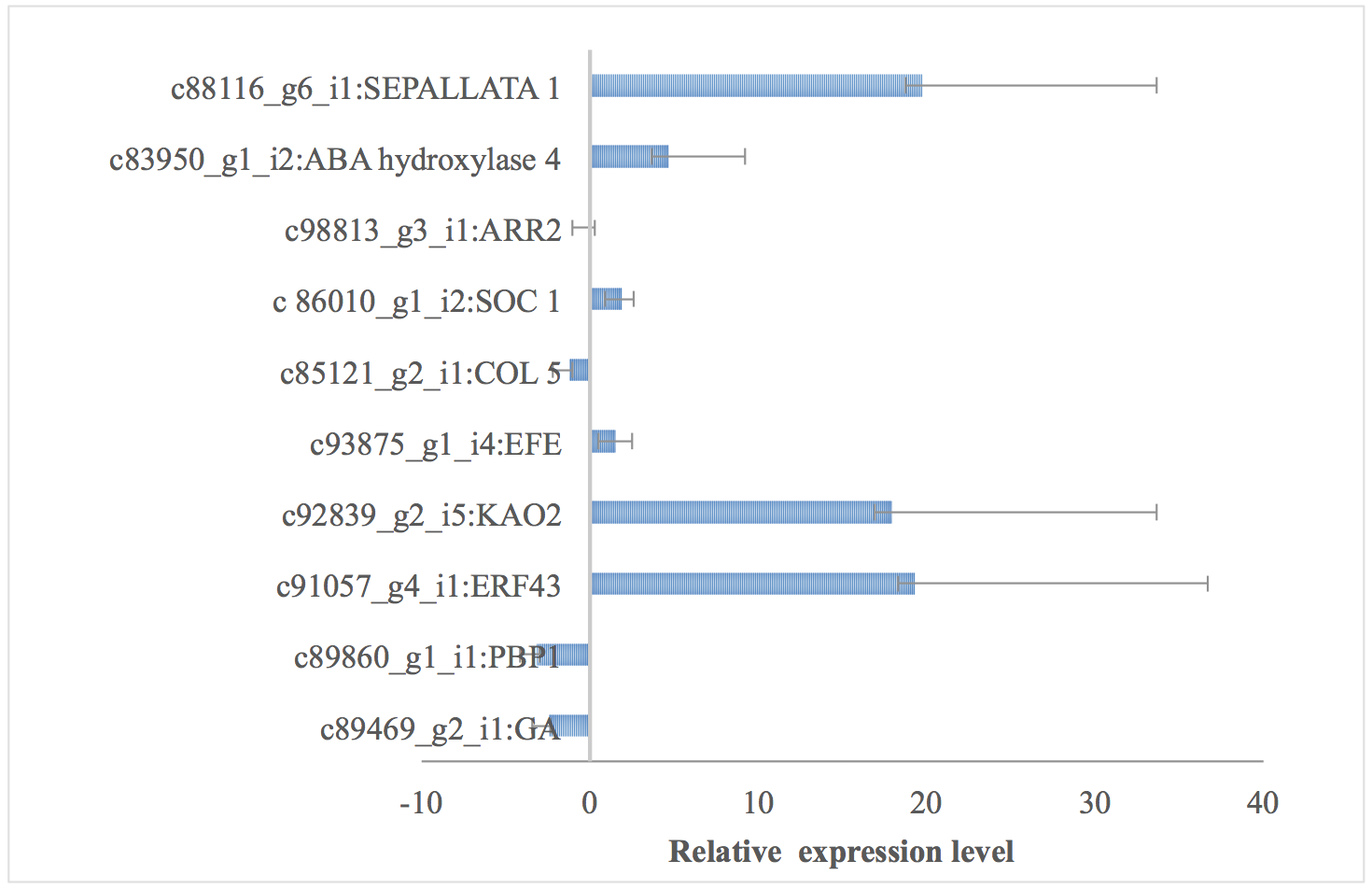

Supplement: Supplementary Figure S5 [file hortres201653-s9.tiff]

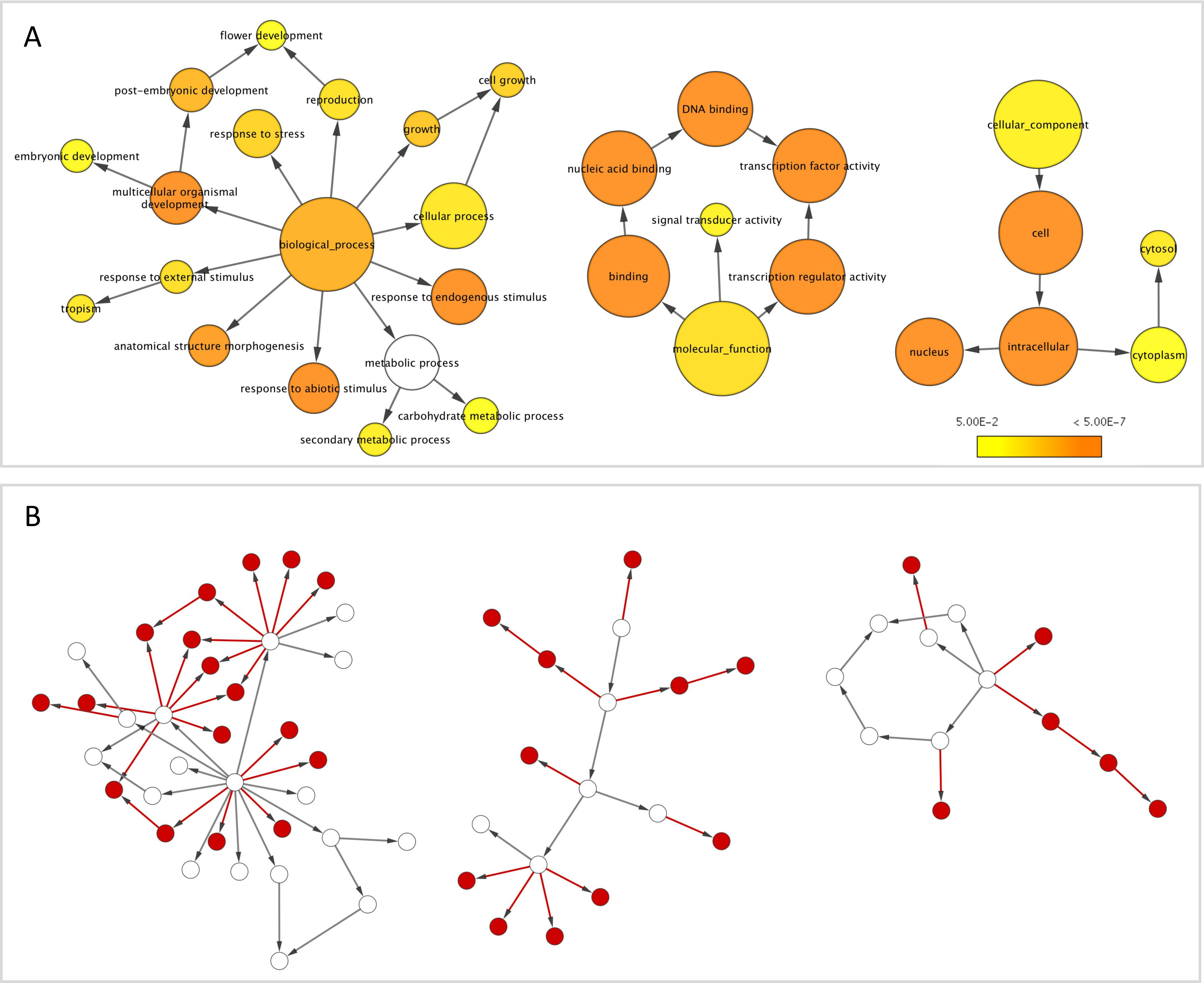

Supplement: Supplementary Figure S6 [file hortres201653-s10.jpg]
