# Supplementary material for: Overexpression of blueberry FLOWERING LOCUS T is associated with changes in the expression of phytohormone-related genes in blueberry plants
Source: Hortic Res. 2016 Oct 26;3:16053–. doi: 10.1038/hortres.2016.53 (PMC5080838; doi:10.1038/hortres.2016.53)
Supplement: Supplementary Legends [file hortres201653-s11.doc]

**Figure S1.** Adventitious shoot regeneration from leaf explants of non-transgenic ‘Aurora’ (**A**) and transgenic ‘VcFT-Aurora’ (**B**) containing an overexpressed *VcFT* after 8 weeks on regeneration medium. *Bars =* 1 cm.

**Figure S2.** GO slims of the differentially expressed genes in leaf tissue of ‘VcFT-Aurora’ plants.

**Figure S3.** Gene networks of phytohormone-related DE genes in leaf tissues of ‘VcFT-Aurora’. Box, a group of overrepresented GO terms (*P* < 0.05) related to phytohormones. Bubble size indicates the frequency of the GO term. Bubble color indicates the *P*-value.

**Figure S4.** Gene networks of phytohormone-related and dwarf-related DE genes in leaf tissues of ‘VcFT-Aurora’. Box I, a group of overrepresented GO terms (*P* < 0.05) related to phytohormones. Bubble size indicates the frequency of the GO term. Box II, a group of overrepresented GO terms (*P* < 0.05) related to plant size. Box III, a group of overrepresented GO terms (*P* < 0.05) related to plant flowering. Bubble color indicates the *P*-value.

**Figure S5. q**RT-PCR analysis of differentially expressed transcripts in leaf tissues of non-transgenic ‘Aurora’ and transgenic ‘VcFT-Aurora’. Eukaryotic translation initiation factor 3 subunit H is the internal control.

**Figure S6.** Gene networks of differentially expressed genes in leaf tissue of ‘VcFT-Aurora’ plants. **A** The network of phytohormone-responsive and dwarf-related genes. **B** Comparison of the gene network of the phytohormone-responsive and dwarf-related genes with that of all DE genes of ‘VcFT-Aurora’. The white nodes are shared by both gene networks, and the red nodes are present only in the gene network for all DE genes. For **A** and **B**, the ontology file of GOSlim_Plants in BiNGO was used to identify overrepresented GO terms (*P* < 0.05).

**Table S1.** Gene Ontology (GO) terms used to search for genes/transcripts related to phytohormones and dwarf plants.

**Table S2.** Primers used for qRT-PCR. FDR (false discovery rate) = 0.05. LogFC: log_2_(fold change) = Log_2_(VcFT-Aurora/Aurora).

**Table S3.** Differentially expressed phytohormone-related genes (compared to non-transgenic 'Aurora') in leaf tissues of 'VcFT-Aurora'. LogFC: log_2_(fold change) =Log_2_(VcFT-Aurora/Aurora).

**Table S4.** Differentially expressed dwarf-related genes in 'VcFT-Aurora' (vs. non-transgenic 'Aurora'). FDR (false discovery rate) = 0.05. LogFC: log_2_(fold change) = Log_2_(VcFT-Aurora/Aurora). *Transcript has more than one annotation.
